# The Effectiveness of Postharvest Processing on Microbiological Safety of Game Meat—A Systematic Review

**DOI:** 10.1111/1541-4337.70420

**Published:** 2026-02-28

**Authors:** Naim Deniz Ayaz, Ali Aydin, Ewa Bilska‐Zając, Raffaella Branciari, Gunita Deksne, Vangelis Economou, Bożena Futoma‐Kołoch, Robert Głogowski, Eduarda Gomes Neves, Famke Jansen, Weronika Korpysa‐Dzirba, Andrea Lauková, Thomai Lazou, Guðný Rut Pálsdóttir, Maria Francesca Peruzy, Petras Prakas, David Ranucci, Rossana Roila, Mirosław Różycki, Selene Rubiola, Ioannis Sakaridis, Madalena Vieira‐Pinto

**Affiliations:** ^1^ Department of Food Hygiene and Technology, Faculty of Veterinary Medicine Kırıkkale University Yahsihan Kirikkale Türkiye; ^2^ Department of Food Hygiene and Technology, Faculty of Veterinary Medicine İstanbul University‐Cerrahpasa Avcılar Istanbul Türkiye; ^3^ Department of Parasitology and Invasive Diseases National Veterinary Research Institute Puławy Poland; ^4^ Veterinary Research Center on Wildlife, Department of Veterinary Medicine University of Perugia Perugia Italy; ^5^ Faculty of Medicine and Life Sciences University of Latvia Riga Latvia; ^6^ Laboratory of Animal Food Products Hygiene—Veterinary Public Health, Faculty of Veterinary Medicine, Faculty of Health Sciences Aristotle University of Thessaloniki, AUTh University Campus Thessaloniki Greece; ^7^ Department of Microbiology University of Wroclaw Wroclaw Poland; ^8^ Department of Animal Breeding and Nutrition, Institute of Animal Sciences Warsaw University of Life Sciences Warsaw Poland; ^9^ Department of Pathology and Molecular Immunology ICBAS ‐ University of Porto Porto Portugal; ^10^ Department of Biomedical Sciences Institute of Tropical Medicine Antwerp Belgium; ^11^ Centre of Biosciences of the Slovak Academy of Sciences Institute of Animal Physiology Košice Slovakia; ^12^ Laboratory of Animal Food Products Hygiene ‐ Veterinary Public Health, School of Veterinary Medicine Aristotle University of Thessaloniki Thessaloniki Greece; ^13^ Institute for Experimental Pathology at Keldur University of Iceland Reykjavík Iceland; ^14^ Department of Veterinary Medicine and Animal Production University of Naples “Federico II” Naples Italy; ^15^ State Scientific Research Institute Nature Research Centre, Vilnius, Akademijos 2 Vilnius Lithuania; ^16^ Department of Preclinical Sciences and Infectious Diseases Poznań University of Life Science Poznan Poland; ^17^ Department of Veterinary Sciences University of Turin Grugliasco, Turin Italy; ^18^ Veterinary Research Institute Hellenic Agricultural Organization ‐ Dimitra, Campus of Thermi Thessaloniki Greece; ^19^ Veterinary and Animal Research Centre (CECAV), Associate Laboratory for Animal and Veterinary Sciences (AL4AnimalS) University of Trás‐os‐Montes and Alto Douro (UTAD) Vila Real Portugal

**Keywords:** biological hazards, deer, foodborne pathogens, game meat products, meat processing, meat safety, venison, wild boar

## Abstract

The rising global consumption of game meat has highlighted gaps in the management of biological hazards associated with its production and consumption, and the safety of processed game meat products remain insufficiently addressed. Therefore, there is a need for research evaluating the effectiveness of processing and preservation methods in reducing microbiological risks. Thus, a systematic review adhering to the Preferred Reporting Items for Systematic Review and Meta‐Analysis (PRISMA) guidelines was conducted. The review yielded 65 records detailing the decrease or inactivation of microbiological foodborne pathogens in game meat treated with various processes. Most records focused on bacterial hazards, particularly *Listeria monocytogenes*, *Salmonella* spp., and pathogenic *Escherichia coli*, while only one paper specifically addressed viral concerns, notably hepatitis E virus in wild boar meat products. *Trichinella* spp. emerged as the most referenced parasite, cited in 11 records. Refrigeration and freezing are commonly employed preservation methods but they may not control all hazards, including freeze‐resistant parasites (e.g., *Trichinella nativa*) and psychrotrophic bacteria capable of growing at low temperatures. Curing and fermenting, although generally resulting in microbiologically safe ready‐to‐eat products, showed limited efficacy against certain parasites and bacteria, including Shiga toxin‐producing *Escherichia coli*. Although thermal processing is well known to achieve broad‐spectrum pathogen inactivation, its systematic evaluation as a controlled intervention specifically for game meat remains limited in the scientific literature. Alternative processing methods such as marinating and the use of natural antimicrobials have been minimally studied in game meat. The lack of standardized protocols and insufficient methodological detail across many studies hinder a proper characterization of the hazards involved.

## 1 Introduction

Game meat occupies a historically and culturally significant place around the world. Hunting has long‐shaped social identity, food traditions, and the way humans relate to nature (Ljung et al. 2012). In Europe, for example, it has traditionally been a symbol of prestige, land stewardship, and respect for seasonal and ecological rhythms (Niewiadomska et al. 2020). These European traditions mirror global patterns. In North and South America, indigenous groups rely on hunting to maintain cultural continuity (Hedman et al. 2020; Ponta et al. 2019), while in Africa, communities integrate wild animals into food security strategies and conservation‐linked game ranching (Taylor et al. 2020). In Asia and Oceania, hunting remains a central component of local customs, ecological management and subsistence practices (Arobaya et al. 2021; Bray et al. 2018; Gressier 2016). Game meat acts as a cultural link between traditional lifestyles and modern society across continents (Hoffman and Wiklund 2006). As such, game meat serves as both a gastronomic delight and a symbol of heritage, linking contemporary culinary practices with historical traditions that continue to shape European cuisines today (Needham et al. 2023). In recent years, demand for game meat has increased due to consumers' perception of such products as organic and free of synthetic inputs and as an alternative to industrial, large‐scale livestock farming (Hoffman and Wiklund 2006; Niewiadomska et al. 2020; Tomić Maksan et al. 2025). The high quality of game meat results from various factors, such as the animals’ slow growth in natural and wild environments, their diet based on natural food sources, and the absence of both pharmacological interventions and stress typical for farmed animals (Babicz et al. 2018).

The animal species considered as game meat around the world can vary, as shown in Table 1. In general, the species hunted only for self‐consumption or black‐illegal market are not defined as game meat but as “wildmeat” or “bushmeat” (Lindsay et al. 2013; Pangau‐Adam et al. 2012), and were not considered in the present review.

**TABLE 1:** Game meat species considered in the review, grouped by taxonomic category, and main geographic distribution.

Species	Scientific names	Main geographic area	References
**Ungulates**			
Red deer	*Cervus elaphus*	Asia, Europe, Oceania, North America	de Oliveira et al. 2025 ; Mahmut et al. 2002; Verkhoturov et al. 2022
Elk /Wapiti	*Cervus elaphus nelsoni*	North America	Hoffman and Wiklung 2006
Sika deer	*Cervus nippon*	Asia	Miyata et al. 2024
Sambar deer	*Cervus unicolor*	Oceania	Hampton et al. 2023
White‐tailed deer	*Odocoileus virginianus*	Europe, North America, South America	Rankins et al. 2023; Sauvala et al. 2023
Black‐ tailed deer	*Odocoileus hemionus*	North America	Hedman et al. 2020
Fallow deer	*Dama dama*	Europe, Oceania, North America, South America	Esattore et al. 2022; Hampton et al. 2023
Roe deer	*Capreolus capreolus*	Asia, Europe	Avagnina et al. 2012; Choi et al. 2013
Chital deer	*Axis axis*	Oceania, North America, South America	Fernández et al. 2021; Hedman et al. 2020; Hampton et al. 2023
Reindeer	*Rangifer tarandus*	Asia, Europe, North America	Andronov et al. 2022;
Moose	*Alces alces*	Asia, Europe, North America	Jensen et al. 2018; Jensen et al. 2020
Chamois	*Rupicapra rupicapra*	Europe	Avagnina et al. 2012
Springbok	*Antidorcas marsupialis*	Africa	North and Hoffman 2015
Kudu	*Tragelaphus strepsiceros*	Africa	Hoffman et al. 2009a
Impala	*Aepyceros melampus*	Africa	Hoffman et al. 2009a
Eland	*Taurotragus oryx*	Africa	Needham et al. 2019
Gemsbuck	*Oryx gazella*	Africa	Hoffman et al. 2020
Blesbuck	*Damaliscus dorcas phillipsi*	Africa	Hoffman et al. 2008
Black wildebeest	*Connochaetus gnou*	Africa	Hoffman et al. 2009b
Zebra	*Equus quagga burchellii*	Africa	Hoffman et al. 2016
Giraffe	*Giraffa giraffa angolensis*	Africa	Hoffman et al. 2022
Muskox	*Ovibos moschafus*	Europa, North America	Amouei et al. 2025
Mouflon	*Ovis mosimon*	Asia, Europa	Amouei et al. 2025
Wild boar/Feral hog	*Sus scrofa*	Asia, Europe, Oceania, North America, South America	Lestingi 2023; Markov et al. 2022; Rostami et al. 2017b
Wharthog	*Phacochoerus africanus*	Africa	Rudman et al. 2018
**Lagomorphs and rodents**			
European wild rabbit	*Oryctolagus cuniculus*	Europe	Marín‐García and Llobat 2021
European hare	*Lepus europaeus*	Europe, South America	Bonino et al. 2010; Razmaitė and Šiukščius 2023;
Mountain hare	*Lepus timidus*	Asia, Europe	Reid 2010; Tatarinova et al. 2021
Nutria/Coypu	*Myocastor coypus*	South America, Europe	Saadoun and Cabrera 2019; Slováček et al. 2024
Beaver	*Castor fiber*	Europe	Slováček et al. 2024
Capybara	*Hydrochoerus hydrochaeris*	South America	Nogueira‐Filho and da Cunha Nogueira 2018
**Avian**			
Mallard	*Anas platyrhynchos*	Europe, Asia	Janiszewski et al. 2018; Sauvala et al. 2021;
Woodpigeon	*Columba palumbus*	Europe, Asia	Kokoszyński et al. 2020; Sauvala et al. 2021
Pheasant	*Phasianus colchicus*	Europe, Asia	Amaral et al. 2015; Sandakova et al. 2021;
Common quail	*Coturnix coturnix*	Europe	Amaral et al. 2015
Stubble quail	*Coturnix pectoralis*	Oceania	Hampton et al. 2022
Teal	*Anas crecca*	Europe	Sauvala et al. 2021
Partridge	*Perdix perdix*	Europe	El‐Ghareeb et al. 2009
Red‐legged partridge	*Alectoris rufa*	Europe	Sánchez‐Cano et al. 2024
Chukar partridge	*Alectoris chukar*	Asia	Abd Rabou 2022
Egyptian goose	*Alopochen aegyptiacus*	Africa	Abd El Rahman et al. 2023; Geldenhuys et al. 2014
Guinea fowl	*Numida meleagris*	Africa	Geldenhuys et al. 2014
Pintail	*Anas acuta*	Africa	Abd El Rahman et al. 2023
Shoveler	*Spatula clypeata*	Africa	Abd El Rahman et al. 2023
Eurasian wigeon	*Mareca* *Penelope*	Africa	Abd El Rahman et al. 2023
**Others**			
Bears	*Ursus arctos*	Asia, Europe, North America	Dalcin et al. 2017; Endo et al. 2025; Kelava Ugarković et al. 2020
	*Ursus thibetanus*	Asia	Endo et al. 2025
Kangaroos	*Macropus* spp.	Oceania	Hampton et al. 2023
	*Osphranter* spp.	Oceania	Hampton et al. 2023
Wallaby	*Notamacropus rufogriseus*	Oceania	Hampton et al. 2023

The widespread belief that wild game is naturally “pure” and free from chemicals can create a misleading sense of safety among consumers. In fact, wild game animals, exposed to a variety of environmental factors, may harbor zoonotic bacteria, parasites, and viruses (Table 2). Indeed, field studies have detected *Salmonella* spp. in wild boar and deer lymph nodes, and a high seroprevalence of *Toxoplasma gondii* in wild boar populations (Paulsen et al. 2012; Weindl et al. 2016; Gil Molino et al. 2019; Barroso et al. 2020). Parasites such as *Trichinella* spp. and emerging pathogens like *Alaria alata* can persist in muscle tissues, posing health risks if the meat is consumed undercooked or raw (Pozio 2007; Korpysa‐Dzirba et al. 2021). Additionally, hepatitis E virus (HEV) RNA has been found in game meat, highlighting the potential for viral transmission even through minimally processed meats (Li et al. 2005). A crucial factor in ensuring the safety of venison lies in the processing steps carried out after shooting—from the initial handling of the carcass, through evisceration to refrigeration, storage, and meat processing such as maturation, freezing, heat treatment, curing, fermentation, and smoking. When performed correctly, each of these stages can significantly prevent or reduce the presence of pathogens in the meat (Branciari et al. 2020; Di Gioia et al. 2016; Fraqueza et al. 2021; Ježek et al. 2020). However, the absence of standardized handling protocols, combined with limited awareness of associated risks among those involved in subsistence or small‐scale venison harvesting and processing, remains a significant problem. Therefore, ensuring food safety remains a major challenge in the game meat supply chain (Needham et al. 2023). This topic forms part of an international COST Action (CA22166 Safety in the Game Meat Chain‐SafeGameMeat) that focuses particularly on identifying and assessing known and emerging chemical and biological risks of regional, national or global importance that pose a hazard to human health when consuming game meat. In this context, identifying research gaps in the safety of game meat products is crucial.

**TABLE 2:** Biological hazards associated with game meat, host species, and geographic occurrence.

Hazard group	Pathogen/organism	Game species	Geographic region	References
Bacteria	*Campylobacter* spp.	Warthog, antelope	Africa	Mpalang et al. 2013
		Wild boar, deer	Asia	Magwedere et al. 2013; Asakura et al. 2017
		Wild boar, deer, game birds	Europe	Díaz‐Sánchez et al. 2013; Stella et al. 2018; Sauvala et al. 2023
	*Clostridium botulinum*	Deer	North America	Midura et al. 1972
	*Escherichia coli*/ Shiga‐like toxin‐producing *Escherichia coli*	Warthog, antelopes	Africa	Mpalang et al. 2013
		Wild boar, deer	Asia	Magwedere et al. 2013; Díaz‐Sánchez et al. 2013; Asakura et al. 2017
		Deer, wild boar	Europe	Mateus‐Vargas et al. 2017; Ziomek et al. 2021; Smith‐Palmer et al. 2018
		Deer, elk	North America	Keene et al. 1997
		Kangaroo	Australia/Oceania	Eglezos et al. 2007
	*Listeria monocytogenes*	Deer, wild boar	Europe	Membré et al. 2011; Fredriksson‐Ahomaa et al. 2022
		Kangaroo	Australia/Oceania	Eglezos et al. 2007
	*Mycobacterium bovis*	Kudu, antelope	Africa	Van der Merwe and Michel 2010
		Wild boar	Europe	Clausi et al. 2021
	*Salmonella* spp.	Warthog, antelope	Africa	Mpalang et al., 2013
		Reindeer	Arcitic region/ Syberia	Aschfalk et al. 2002
		Wild boar, deer	Asia	Magwedere et al. 2013; Díaz‐Sánchez et al. 2013; Asakura et al. 2017
		Kangaroo, wallaby	Australia/Oceania	Potter et al. 2011
		Wild boar, red deer, roe deer	Europe	Paulsen et al. 2012; Weindl et al. 2016
		Deer, elk	North America	Díaz‐Sánchez et al. 2013
		Capybara	South America	Farikoski et al. 2019
	*Staphylococcus aureus*	Wild boar	Europe	Łepecka et al. 2023
		Various mammals	South America	Ramos and Cunha 2024
	*Yersinia enterocolitica*	Wild boar	Europe	Avagnina et al. 2012; Sannö et al. 2014
Parasites	*Alaria alata*	Wild boar	Europe	Korpysa‐Dzirba et al. 2021; González‐Fuentes et al. 2015
	*Fasciola gigantica*	Antelope	Africa	Nukeri et al. 2022
	*Sarcocistys* spp.	Sika deer	Asia	Honda et al. 2018
		Macropods	Australia/ Oceania	Munday et al. 1978
		Cervids, Wild boar	Europe	Luzón et al. 2015; Korpysa‐Dzirba et al. 2024
	*Taenia* spp.	Camelids	South America	Cañal and Beltrame 2002
	*Toxoplasma gondii*	Antelope	Africa	Bokaba et al. 2022; Račka et al. 2024
		Bear	Arcitic region/ Syberia	Pilfold et al. 2021
		Wild boar, deer	Asia	Saito et al. 2021
		Kangaroo	Australia/ Oceania	Borkens 2021
		Deer, wild boar	Europe	Paulsen et al. 2012; Weindl et al. 2016
		Deer	North America	Cook et al. 2000
		Capybara, deer	South America	Truppel et al. 2010
	*Trichinella nativa*	Bear	Arcitic region/ Syberia	Oksanen et al. 2022
		Bear	North America	CDC 2004; Gari‐Toussaint et al. 2005; McIntyre et al. 2007; Cash‐Goldwasser et al. 2024
	*Trichinella britovi; Trichinella. spiralis*,	Wild boar	Europe	Pozio et al. 2007; Turiac et al. 2017; Pavic et al. 2020; Peju et al. 2023
Viruses	Hepatitis E virus	Bear, deer	Arcitic region/ Syberia	Keatts et al. 2021
		Deer	North America	Akpoigbe et al. 2023
	Hepatitis E virus genotype 4	Wild boar, deer	Europe	Li et al. 2005; Montone et al. 2019
	HEV‐like viruses	Wild boar, deer	Asia	Wang et al. 2025

This review aims to critically evaluate the literature on the presence of biological hazards (foodborne bacteria, viruses, and parasites) in processed game meat, given that the current methods used to reduce these biohazards during processing have not been fully investigated in these meats. This could help risk assessments, evaluate consumer exposure, and identify practical directions to improve technological practices that can enhance the safety of game meat, while respecting tradition. For this reason, only processing techniques adopted for game meat were considered, rather than being compared with those used for livestock meat products. Both traditional and modern pathogen control techniques are analyzed, including effectiveness against bacterial, viral, and parasite hazards. The importance of risk awareness, maintaining full process oversight, and educating producers and consumers was emphasized. All the terms adopted in this review paper are available as supporting information (Supporting Information 1).

## 2 Overview of Game Meat Processing: Meat Preparation and Meat Products

### 2.1 Fresh Cuts and Meat Preparation (Steaks, Chops, and Roasts)

To ensure that a carcass meets appropriate hygienic standards, different operations can be applied during slaughter and dressing. International guidance documents, such as the Codex Code of Hygienic Practice for Meat, reference measures including potable‐water carcass washing (spray or immersion), immersion chilling, proper management of scalding water, and trimming of visible contamination, all aimed at maintaining contamination at the lowest practicable level (CXC 58–2005). European regulations give limited treatment options on game carcasses: the water used for washing carcasses must be potable, while the use of chemical decontaminants on carcasses is generally restricted and subject to national regulations (EC Reg. 853/2004; European Parliament and Council 2004). Additionally, steam pasteurization is an approved physical decontamination method in the EU, where potable water is used to generate steam applied under controlled conditions to carcasses without causing irreversible changes to the meat (EFSA 2010). In other jurisdictions, such as the United States, the USDA‐FSIS authorizes the use of several antimicrobial agents for meat decontamination, including lactic acid (up to 5% on carcasses), buffered organic acids, the bacteriocin nisin (GRAS‐listed), and lactoferrin, reflecting a broader set of permitted interventions (Yim 2025). In this context of comparatively narrower EU options, recent scientific evaluations and EFSA opinions have recognized the potential use of alternative agents, such as weak organic acids, which may be incorporated into HACCP systems at critical control points under strict regulatory conditions (EFSA 2022).

In general, cold temperatures and freezing represent the most common methods for preserving game meat, preventing spoilage, and reducing the risk of bacterial and parasitic foodborne illnesses (Paulsen and Winkelmayer 2004). Rapidly lowering the temperature of wild game to below 4.4°C (40°F) is essential to inhibit bacterial proliferation and maintain meat quality (Schmutz et al. 2007). In line with this principle, general meat hygiene standards—not limited to game species—highlight the importance of prompt carcass cooling after slaughter. Several countries suggest that carcasses should be chilled at temperatures between 0°C and 4°C for 24–48 h postmortem to ensure adequate thermal stabilization. Furthermore, Europe, Canada, and South Africa indicate that the internal temperature of carcasses should be decreased to below 7°C before processing into primal and subprimal cuts. As part of this broader framework, refrigeration may be carried out directly at collection centers on skin‐on carcasses, with the cold chain maintained until arrival at the game handling establishment. In some countries, such as the Czech Republic, national legislation also permits refrigerated skin‐on game intended for direct sale to be stored for up to 15 days after hunting at temperatures between 0°C and 1°C (Zhang et al. 2019; EU Reg. 853/2004). The optimal approach involves implementing the cooling process on the day of the hunt, with the carcass being transferred to a designated cooler (Anonymous 2025). Particular care should be taken to keep the carcass cool or refrigerated, especially during the hot hunting season (Schmutz et al. 2007).

The storage temperature appears to impact the hygiene quality of the meat, which may be due to the effect of cooling conditions on contamination levels (Membré et al. 2011). If cooling is insufficient, microbial growth can occur. This may lead to the surface of the meat or carcass accumulating high levels of pathogens and spoilage microorganisms, ultimately affecting meat quality and safety.

Hunters who sell game to wholesalers or game processing companies are considered food business operators and are responsible for food safety. They are subject to the same hygiene regulations as game processing plants or other food business operators. Any failure to respect these procedures may result in impaired quality and violation of the safety of the game meat. However, if the appropriate storage regime is observed, meat of extremely good quality posing no risk to the consumer can be obtained and distributed even after 15 days of storage (Borilova et al. 2016).

Fresh cuts of game meat are typically produced by butchering the carcass into primal cuts, which are further divided into steaks, chops, and roasts, depending on the species and the desired product. For example, venison might be cut into steaks and roasts, while wild boar might be prepared as chops. These cuts require minimal processing beyond cleaning and portioning, though some may be marinated or seasoned before sale. The preservation of meat quality is a primary consideration, with factors such as aging, packaging, and storage temperature affecting the final product's sensory and nutritional properties. Game meat is also usually available frozen. When prepared from fresh cuts, fallow deer mince retains its red color better during retail display than when processed from frozen or thawed meat (Chakanya et al. 2017; Djekic et al. 2023).

### 2.2 Cured, Fermented, and Smoked Products (Sausages, Jerky, and Pâtés)

Curing and smoking are age‐old techniques used to extend the shelf life of game meat and enhance its flavor. Sausages, jerky, and pâtés are common examples of cured and smoked products (Wójciak et al. 2024). These products undergo processes like salting, drying, fermentation, and smoking, which not only preserve the meat but also impart unique flavors and textures (Kononiuk and Karwowska 2020; Vargas‐Ramella et al. 2021). Sausages are typically made by grinding the meat and mixing it with fat, spices, and sometimes preservatives, followed by stuffing the mixture into casings. The sausages may undergo smoking, drying, or fermenting to improve shelf life (Ranucci et al. 2019; Chakanya et al. 2020). For example, the basic ingredients of “salame di daino” (dry fermented sausage produced in Italy) are lean fallow deer meat, lean pork, pork fat, salt, pepper, garlic, white wine and ascorbic acid. No milk, milk powder, sugars, or additives are added (Cenci Goga et al. 2012). Jerky, a dried product, is often prepared by marinating thin slices of meat in salt and spices before drying it, which both preserves and concentrates the meat's flavor (Nummer et al. 2004). Pâtés involve grinding or pureeing meat, often with added fat, herbs, and spices, which is then cooked or sometimes smoked. Pâté is typically a soft spread, with a creamy texture that contrasts the more robust characteristics of the meat (Adenuga et al. 2025).

### 2.3 Ready‐to‐Eat Meals (Precooked Dishes for Convenience)

The demand for convenience foods has led to the development of ready‐to‐eat meals that use game meat. These precooked dishes are designed to save time and effort for the consumer while offering a more natural alternative to conventional processed foods. The production of ready‐to‐eat meals may involve precooking the meat, then combining it with other ingredients such as vegetables, grains, and sauces. These meals are often vacuum‐sealed or canned to ensure their shelf life without compromising quality.

Precooked game meat meals are an emerging segment of the market, particularly in regions with high hunting and wild game consumption. Research on these products has examined various preservation methods, such as sous vide cooking and advanced freezing techniques, which help retain nutritional value and sensory quality (Baldwin 2012; Kačániová et al. 2024a, 2024b).

## 3 Material and Methods

**FIGURE 1:**
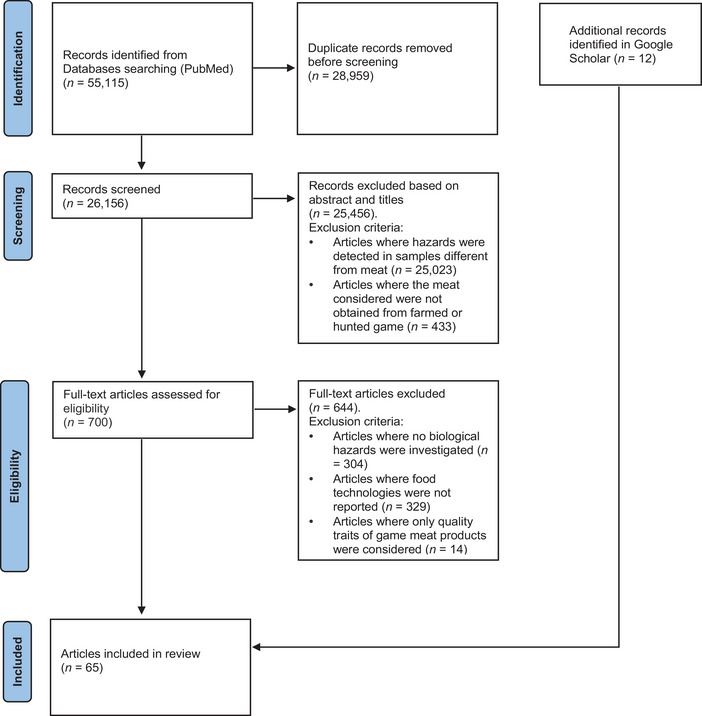
PRISMA diagram describing the records selection process.

Finding pertinent research on the impact of various processing methods used in the food industry on the existence (survival) or inhibition of microbiological foodborne pathogens' growth in game meat products was the aim of the search. For this reason, a systematic review was conducted adopting the Preferred Reporting Items for Systematic Review and Meta‐Analysis standards (Page et al. 2021). PubMed was used as the primary database to find pertinent investigations, and Google Scholar was used as a secondary database to find records that would not have been located in the prior platform. The search includes all the research articles, case reports, and review papers available in the databases considered until January 2025. The keywords adopted and the selection methods, including data mining, are reported in Table 3 and Figure 1, respectively.

**TABLE 3:** Keywords adopted for the systematic review on game meat products' safety.

Levels	Keywords
Level 1: Game meat terms	Game meat OR Venison OR Deer OR Wild boar
Level 2: Food safety terms	Food safety OR Foodborne pathogens OR Foodborne disease OR Foodborne illness
Level 3: Microbiological terms	Microbiology OR Bacteria OR Virus OR Parasite OR Salmonella OR Campylobacter OR Listeria OR Mycobacterium OR Brucella OR Escherichia coli OR Clostridium OR Bacillus OR Hepatitis OR Trichinella OR Toxoplasma
Level 4: Food technology terms	Product OR Process OR Maturing OR Aging OR Heating OR Cooking OR Freezing OR Marinating OR Natural substance OR Extracts OR Antimicrobial OR Smoking OR Salting OR Curing OR Fermented OR Hurdle Technology OR Sous vide OR Sausages OR Salami OR Ham

The selection of the records was performed with the following exclusion parameters (Figure 1):

- papers where hazards were detected in samples different from meat (e.g., blood, fecal material);
- papers where the meat considered were not obtained from farmed or hunted game (e.g., bushmeat, meat of wildlife hunted for human consumption, particularly in parts of Africa, Asia, and South America, commonly harvested through unregulated or subsistence hunting practices, and marine mammals such as seals);
- papers where no biological hazards were investigated (e.g., chemical hazards, spoiling bacteria, technological bacteria);
- papers where food processing technologies were not mentioned (e.g., carcasses, fresh meat);
- papers where only quality traits of game meat products were considered (e.g., color, texture).

After duplicate records were erased, a total of 26,157 PubMed records were obtained using the keywords taken into consideration. Therefore, data mining was required to identify the publications that were completely consistent with the research's objective, excluding those that had nothing to do with the processing of game meat or its products combined with biological hazards reduction or elimination (Figure 1). One record was removed because only the abstract was in English language (Yamamoto et al. 2022). Not all of the articles identified in the databases for the systematic review of game meat referred to the origin of the raw material, that is, whether it came from hunted or farmed game. When in doubt, all articles matching the keywords were included. Information on the decrease or inactivation of microbiological foodborne pathogens in game meat treated with at least one of the processes (see Table 4) was available in 53 PubMed records obtained through data mining, and an additional 12 were identified via Google Scholar, for a total of 65 records.

**TABLE 4:** Details on the class of records obtained in the systematic review from the selected database.

Classes	Objects	Number
Class 1: Kind of record	Scientific papers	46
	Case studies	9
	Review papers	10
Class 2: Kind of hazard^a^	Bacteria Virus Parasites	47 2 18
Class 3: Kind of process^b^	Cold temperatures High temperatures Curing and fermentation Others	26 11 29 7
Class 4: Details in process description and product parameters^c^	No description Partial description Full description Final product description	27 4 19 5
Class 5: Detail in the process efficacy against hazards	Presence/absence Specific	52 13

^a^ One review paper refers mainly to bacteria, but mentions also parasites and viruses, and was considered in the count.

^b^ Eight records describe more than one process, and this was considered in the count.

^c^ The review papers were not considered for this class.

The records were then sorted in classes according to:

- The kind of records: Scientific papers, review articles, case studies.
- The kind of biological hazards: Bacteria, viruses, parasites (including protozoa and helminths).
- The kind of process: Cold temperature (maturing, aging, freezing), high temperature (heating, cooking), curing/fermenting process, other processes (e.g., smoking, marinating, or the use of antimicrobial plant extracts).
- The details in process description: No description of process and parameters, description only of parameters of the final products, partial description of process and parameters, full description of the process and parameters (e.g., parameters considered pH, temperature, time).
- The detail in the process efficacy against hazards: Presence/absence in the products, specific studies on inactivation or reduction of biological hazards.

## 4 Results

The keyword combinations and data mining made it possible to obtain 65 records from the selected databases, covering all three types of records. In particular, 46 Scientific papers, 10 review articles, and 9 case studies. Among biological hazards, most of the records were on bacteria and on curing, fermentation and cold temperature use in game meat products (Table 4). According to the bacterial hazards considered, *Escherichia col*i, including Shiga toxin‐producing *Escherichia coli* (STEC), S*almonella* spp. and *L. monocytogenes* were mentioned in 27, 22, and 22 papers, respectively. Only one paper was specific to viruses, specifically regarding HEV in wild boar meat products. The most referred parasite was *Trichinella* spp. (11 records), in particular referring to case study papers. Limited information is generally provided on product description, and other parameters considered as intrinsic and extrinsic factors for biological hazards controls (such as pH, activity water (aw), salt content, temperature, time), with only 19 records that describe them. Some of the records investigate only the absence of the hazards in the final products, not giving clear evidence of their presence in the raw game meat used during production, and in some products (e.g., sausages and salami) different percentages of meat from livestock animal (pork mainly) were also used in the recipes.

### 4.1 Cold Temperature and Freezing

The storage temperature appears to impact the hygiene quality of the meat, which may be due to the effect of cooling conditions on contamination levels (Membré et al. 2011). If cooling is insufficient, microbial growth can occur. This may lead to the surface of the meat or carcass accumulating high levels of pathogens and spoilage microorganisms, ultimately affecting meat quality and safety. In this context, more than 20 relevant zoonotic pathogens have been reported in game meat (Ruiz‐Fons 2017). *L. monocytogenes*, *Yersinia* spp., *Salmonella* spp., pathogenic *E. coli*, and *Campylobacter* spp. are foodborne pathogens often associated with inadequate hunting hygiene (Hedman et al. 2020). In addition, *L. monocytogenes* and *Yersinia* spp. are particularly problematic as they can grow during storage under vacuum at temperatures below 6°C (Sauvala et al. 2023). Epidemiological studies conducted worldwide have revealed the prevalence *of L. monocytogenes* (Kanai et al. 1997; Membré et al. 2011; Fredriksson‐Ahomaa et al. 2022), *Yersinia enterocolitica* (Avagnina et al. 2012; Sannö et al. 2014), *Salmonella* spp. (Gauthier et al. 2010; Díaz‐Sánchez et al. 2013; Mirceta et al. 2017), *E. coli* (Mateus‐Vargas et al. 2017; Ziomek et al. 2021) including shiga‐like toxin‐producing *E. coli* (STEC; Magwedere et al. 2013; Asakura et al. 2017; Smith‐Palmer et al. 2018), and *Campylobacter* spp. (Díaz‐Sánchez et al. 2013; Stella et al. 2018; Sauvala et al. 2023) in different game meats. Indeed, numerous publications have been reported regarding outbreaks caused by different serotypes of STEC (O157:H7, non‐O157:H7) in deer meat (Keene et al. 1997; Otani 1997; Rabatsky‐Ehr et al. 2002; Rounds et al. 2012; Smith‐Palmer et al. 2018). Therefore, for game meat products that cause outbreaks, good hygiene and proper cooking practices by consumers are important control measures to manage the risk of pathogens (Fredriksson‐Ahomaa et al. 2022).

There are relatively few studies on bacterial contamination of game meat stored under cold and frozen conditions worldwide (Tables 5 and 6). The majority of the articles focused on large game, particularly wild boar and deer, while a few studies examined small wild game, such as rabbits and wild birds. As most of the research was reported in Europe, studies on wild game from other continents would be desirable. Several publications indicate that there is still a need for improvement regarding meat hygiene, and therefore safety, under chilling and frozen conditions, particularly for deer, wild boar, and small wild game meat (Gomes‐Neves et al. 2021; Peruzy et al. 2019; Membré et al. 2011).

**TABLE 5:** Studies on bacterial contamination of chilled game meat.

Condition	Species	Country	Bacteria (mean log_10_ cfu/g − cfu/cm^2^ or positive/total number*)					References
			APC	EC (STEC)	ENT	*C. perfringens*	*S. aureus*/CPS	
Chilled (4°C)	Red deer (*n* = 120)	Japan	115/120*	18/120* (1/120)	n.d.	n.d.	7/120*	Asakura et al. 2017
	Wild boar (*n* = 128)		127/128*	64/128* (0/128)	n.d.	n.d.	19/128*	
Chilled (4°C)	Chamois (*n* = 65)	Italy	3.23	n.d.	1.30	n.d.	n.d.	Avagnina et al. 2012
	Roe deer (*n* = 61)		3.46	n.d.	2.47	n.d.	n.d.	
	Red deer (*n* = 61)		3.31	n.d.	1.70	n.d.	n.d.	
	Wild boars (*n* = 65)		4.61	n.d.	3.00	n.d.	n.d.	
Chilled (4°C)	Red deer (*n* = 271)	Spain	n.d.	19/271*	n.d.	n.d.	n.d.	Diaz‐Sanchez et al. 2013
	Wild boar (*n* = 310)		n.d.	12/310*	n.d.	n.d.	n.d.	
Chilled (4°C)	White‐tailed deer (*n* = 3)	United States	n.d.	3/3	n.d.	n.d.	n.d.	Magwedere et al. 2013
	Red deer (*n* = 20)		n.d.	2/20	n.d.	n.d.	n.d.	
	Reindeer (*n* = 2)		n.d.	0/2	n.d.	n.d.	n.d.	
	Wild boar (*n* = 2)		n.d.	0/2	n.d.	n.d.	n.d.	
	Bison (*n* = 24)		n.d.	3/24	n.d.	n.d.	n.d.	
	Wild Rabbit (*n* = 4)		n.d.	0/4	n.d.	n.d.	n.d.	
Chilled (4°C)	Wild boar (*n* = 210)	Serbia	5.4	n.d.	3.8	n.d.	n.d.	Mirceta et al. 2017
Chilled (4°C)	Wild boar (*n* = 37)	Italy	4.67	n.d.	2.60	n.d.	n.d.	Orsoni et al. 2020
Chilled (5°C)	Wild boar (*n* = 120)	Italy	3.66	n.d.	2.05	n.d.	n.d.	Ranucci et al. 2021
Chilled (1°C–8°C)	White‐tailed deer (*n* = 100)	Finland	4.5	0.7	1.5	n.d.	n.d.	Sauvala et al. 2019
	Moose (*n* = 100)		4.2	1.2	2.6	n.d.	n.d.	
Chilled (4°C)	Wild boar (*n* = 62)	Italy	3.21	1.31	1.32	n.d.	n.d.	Stella et al. 2018

Review papers excluded.

Abbreviations: APC, aerobic plate count; *C. perfringens*, *Clostridium perfringens* count; CPS, coagulase positive *Staphylococcus* count; EC, *Escherichia coli* count (STEC, shiga toxin‐producing *E. coli*); ENT, *Enterobacteriaceae* count; (F), female; (M), male; *S. aureus*, *Staphylococcus aureus* count.*: positive samples/total number of samples

**TABLE 6:** Studies on bacterial contamination of frozen game meat.

Condition	Specie	Country	Bacteria (mean log_10_ cfu/g − cfu/cm^2^ or positive/total number*)					References
			APC	EC (STEC)	ENT	*C. perfringens*	*S. aureus*/CPS	
Frozen (← 20°C)	Pintail (*n* = 24)	Egypt	5.8(B), 5.7(T)	4.4(B), 4.3(T)	4.5(B) 4.5(T)	2.2(B) neg	3.5(B), 3.5(T)	Abd El Rahman et al. 2023
	Shoveler (*n* = 24)		5.5(B), 5.7(T)	3.6(B), 4.1(T)	4.0(B), 4.6(T)	neg	3.4(B), 3.3 (T)	
	Eurasian wigeon (*n* = 12)		4.1(B), 4.5(T)	3.3(B), 3.7(T)	4.2(B), 4.1(T)	neg	neg	
	Egyptian geese (*n* = 8)		4.2(B), 4.7(T)	3.8(B), 3.5(T)	4.7(B), 4.8(T)	neg	1.5(B), neg	
Frozen (← 18°C)	Red Deer (*n* = 48)	Spain	n.d.	4/48*	n.d.	n.d.	n.d.	Díaz‐Sánchez et al. 2012
	Wild boar (*n* = 36)		n.d.	1/36*	n.d.	n.d.	n.d.	
Frozen (Vacuum package, ← 18°C)	Red deer (*n* = 75)	Germany	n.d.	5/75*	n.d.	n.d.	n.d.	Mateus‐Vargas et al. 2017
	Roe deer (*n* = 78)		n.d.	8/78*	n.d.	n.d.	n.d.	
	Wild boar (*n* = 76)		n.d.	4/76*	n.d.	n.d.	n.d.	
Frozen (← 18°C)	House sparrow (*n* = 330)	Tunisia/ Italy	5.71	n.d.	n.d.	n.d.	n.d.	Pasquali et al. 2014
	Starling (*n* = 140)		3.30	n.d.	n.d.	n.d.	n.d.	

Review papers excluded.

Abbreviations: APC, aerobic plate count; (B), breast; *C. perfringens*, *Clostridium perfringens* count; CPS, coagulase positive *Staphylococcus* count; EC, *Escherichia coli* count (STEC, shiga toxin‐producing *E. coli*); ENT, *Enterobacteriaceae* count; *S. aureus*, *Staphylococcus aureus* count; (T), thigh.*: positive samples/total number of samples

It is posited that the game meat will remain safe to consume as long as it is frozen but, after thawing bacteria (including *Mycobacterium bovis*) could be viable and potentially harmful if the cooking process is not optimally performed (Clausi et al. 2021). However, it is recommended that frozen game meat cuts should be consumed within 6–9 months to ensure optimal quality and freshness (Anonymous 2010). Although vacuum packaging and freezing have been shown to effectively extend the shelf life of venison, it is recommended that it should not be stored for more than 1 year to maintain its quality (Sauvala et al. 2023).

Freezing has long been considered a practical strategy for controlling parasitic infections in game meat, especially when immediate cooking is not possible. However, while freezing is effective against certain parasites such as *Trichinella spiralis*, its efficacy is significantly reduced when dealing with freeze‐resistant species (Cash‐Goldwasser et al. 2024). These include *Trichinella nativa*, *Trichinella britovi*, and the T6 genotype—parasites commonly found in wildlife in Arctic and sub‐Arctic regions (Gajadhar and Forbes 2010) but also in Europe (*T. britovi*, Pozio et al. 2007). Studies and outbreak investigations have demonstrated that *T. nativa* larvae can survive in frozen meat for over 110 days (CDC 2023), and that *T. britovi* retains infectivity after 3 weeks at −20°C and even 7 days at −35°C (Pozio and Zarlenga 2005; Pozio 2019). This high level of cold tolerance is believed to be an evolutionary adaptation to the parasites’ natural host environments, which are characterized by prolonged cold temperatures (Pozio 2016). The persistence of these parasites under freezing conditions has critical implications for food safety, particularly for communities and individuals who rely on wild game as a protein source (CDC 2024). Outbreaks of trichinellosis associated with the consumption of frozen and subsequently cooked wild boar and bear meat have been documented in multiple regions, including North America and Europe (CDC 2004; Gari‐Toussaint et al. 2005). Some of these infections have been reported among individuals who consumed thoroughly stewed bear meat, suggesting potential postprocess contamination with raw meat, despite prolonged heating treatment (McIntyre et al. 2007). These outbreaks demonstrate that while freezing is a useful storage technique, it cannot substitute for proper cooking in inactivating freeze‐resistant *Trichinella* spp.

In addition to *Trichinella* spp., other parasites, such as *T. gondii*, pose a significant risk in game meat. Unlike *T. nativa* and *T. britovi*, *T. gondii* tissue cysts are more susceptible to freezing; studies have shown that exposure to −12°C for at least 2 days or −20°C for 3 days can render the parasite inactive in the meat of livestock (Kotula et al. 1991; Kijlstra and Jongert 2008). However, the efficiency of inactivation is influenced by variables such as cyst burden, meat thickness, and the rate of temperature drop during freezing. Therefore, a combination of freezing and thorough cooking is the most reliable method of reducing the transmission of *T. gondii* through food, particularly given the parasite's global prevalence and its ability to cause severe disease in immunocompromised individuals and developing fetuses (Kuruca et al. 2023).

### 4.2 High Temperature and Cooking

Thermal processing remains one of the most traditional and straightforward techniques for inactivating foodborne pathogens and spoilage microorganisms. By applying high temperatures, vital cellular structures are disrupted through denaturation or coagulation, thereby enhancing the safety, quality, and overall hygiene of treated foods (Espinosa et al. 2020; Park et al. 2021). The effectiveness of thermal treatment depends on both the temperature applied and the duration of heating, as well as the inherent heat resistance of the microorganisms. Specifically, the time‐temperature relationship plays a critical role: higher temperatures can achieve effective inactivation in shorter times, whereas lower temperatures require longer exposure (Den Besten et al. 2018; Espinosa et al. 2020). Additionally, the heating process is influenced by factors such as equipment design, heating medium, food size and shape, and product composition and viscosity (Den Besten et al. 2018).

When it comes to game meat and related products, studies examining heat treatment against pathogenic bacteria are comparatively limited (Table 7). Most existing research has focused on the efficacy of sous vide processing—a method involving vacuum‐sealing food and subjecting it to precisely controlled low temperatures (typically ≤60°C) over extended periods. Sous vide is recognized not only for its microbial control potential but also for improving flavor, texture, and nutrient retention (Abel et al. 2020; Romeo et al. 2024). For example, Abel et al. (2020) investigated the thermal inactivation of *L. monocytogenes* in wild boar and roe deer muscles under sous vide conditions, revealing that decimal reduction times (*D*‐values) were significantly influenced by the meat's composition and structure with differences in fat levels (a highly significant correlation between higher fat content and higher *D*‐value were found) and matrix‐related properties (e.g., muscle pH) influencing heat transfer and bacterial survival, accounting for the longer *D*‐values observed in wild boar compared with roe deer. Similarly, Kačániová et al. (2024a, 2024b) demonstrated the reduction of *L. monocytogenes* and *Salmonella enterica* in deer muscles processed sous vide at temperatures between 50°C and 65°C, with effectiveness enhanced by the addition of natural antimicrobial agents such as *Piper nigrum* and *Eugenia caryophyllus* essential oils.

**TABLE 7:** Studies on thermal processing of game meat.

Origin and type of products (samples)	Country of the study	Type of processing and temperatures	Obtained result	References
Wild boar (*n* = 22)	Germany	Microwave heating (90 s)	Complete inactivation of *Alaria alata*	González‐Fuentes 2015
Wild boar(^a^)	Poland	Heating to 72°C (≥2 min)	Recommended to ensure parasite inactivation	Korpysa‐Dzirba et al. 2021
Wild boar (*n* = 8) and roe deer (*n* = 10)	Germany	Sous vide processing (≤60°C, extended periods)	Reduction of *Listeria monocytogenes*	Abel et al. 2020
Sika deer (*n* = 20)	Japan	Heating meat to 70°C (1 min)	Effective inactivation of *Sarcocystis* cysts	Honda et al. 2018
Red deer (*n* = 3; *n* = 1)	Slovakia	Sous vide (50°C–65°C) combined with antimicrobials (*Piper nigrum*, *Eugenia caryophyllus* essential oils)	Reduction of *Listeria monocytogenes* and *Salmonella enterica*	Kačániová et al. 2024a, 2024b
Venison (sausage) (^a^)	United States	Commercial thermal processing (64°C–68°C)	Effective reduction of *Escherichia coli* O157:H7	Roberts and Getty 2011
Game meat (diverse species) (^a^)	United States	≥61°C for ≥3.6 min; FDA recommends 62.8°C with 3‐min rest	Inactivation of *Toxoplasma gondii* cysts	Dubey et al. 1990; Jones and Dubey 2012
Bear meat (*n* = 1)	United States	Cooking ≥74°C (165°F)	Prevention of *Trichinella* infection	Cash‐Goldwasser et al. 2024
African buffalo (*n* = 7) and greater kudu (*n* = 7)	South Africa	Cooking and drying (temperatures unspecified)	Elimination of *Mycobacterium bovis*; partial persistence of nontuberculous mycobacteria	Van der Merwe and Michel 2010

Review papers excluded.

^a^ Number of samples not available.

Beyond sous vide, Roberts and Getty (2011) evaluated a commercial thermal process for acidified venison summer sausage contaminated with *E. coli* O157:H7. Their findings showed robust pathogen reduction at internal temperatures around 64°C–68°C, regardless of sausage fat content or acidulant type, highlighting the consistency of thermal inactivation across different product formulations.

Thermal processing also proves effective against other significant pathogens in game meat. Van der Merwe and Michel (2010) showed that cooking and drying eliminated *M. bovis* in infected African buffalo and greater kudu meat, though nontuberculous mycobacteria were not inactivated in some tissues, indicating the need for further veterinary and public health attention.

Regarding parasites, heat treatment is widely considered the most effective control measure. González‐Fuentes (2020) demonstrated that microwave heating for 90 s successfully inactivated all developmental stages of *A. alata* mesocercariae in wild boar meat. The German Federal Institute for Risk Assessment recommends heating wild boar meat to 72°C for at least 2 min to ensure parasite inactivation (Korpysa‐Dzirba et al. 2021). For *Trichinella* spp., US authorities advise cooking wild game meat to a minimum internal temperature of 74°C (165°F) to prevent infection—a recommendation reinforced by a 2022 outbreak linked to undercooked bear meat (Cash‐Goldwasser et al. 2024). Indeed, when *T. spiralis* larvae were removed from pork chops and cooked in a microwave at 71°C–82°C (2.9–3.1 min) infection in rats occurs (Kotula et al. 1983). Notably, both the CDC and the International Commission on Trichinellosis discourage slow cooking over open fires due to unreliable temperature control and incomplete parasite inactivation (Rostami et al. 2017a).

A European case–control study further identified undercooked game meat as a significant risk factor for *T. gondii* infection during pregnancy (Cook et al. 2000). Research indicates that *T. gondii* tissue cysts become nonviable after exposure to ≥61°C for 3.6 min (Dubey et al. 1990). However, cooking effectiveness can vary based on meat type, cut thickness, and preparation method; grilling, for instance, may fail to achieve uniform temperatures sufficient for parasite inactivation (Kijlstra and Jongert 2008). Despite this, there remains a considerable gap in targeted research on the thermal inactivation of *T. gondii* specifically in game meat. Current US FDA guidelines recommend cooking meat to 62.8°C with a 3‐min rest, increasing to 71°C for vulnerable groups such as pregnant women (Food Safety and Inspection Service, USDA, News and Events 2011).

Lastly, avoiding the consumption of raw or undercooked game meat is critical to prevent enteric infections caused by *Sarcocystis* species. Studies on sika deer have shown that heating meat to 70°C for 1 min effectively inactivates *Sarcocystis* spp. cysts (Honda et al. 2018).

In summary, while specific studies on heat treatment in game meat are relatively sparse, existing evidence strongly supports that high‐temperature thermal processing—whether via sous vide, conventional cooking, or drying—is effective in inactivating a broad spectrum of bacterial pathogens and parasites. Given the variability in meat types and processing methods, proper heat treatment remains essential for ensuring the microbiological safety of game meat products. Further research is warranted to refine thermal inactivation parameters tailored to diverse game meat species and cuts.

### 4.3 Curing and Fermenting

Regarding foodborne bacterial pathogens, available data suggest that curing of game meat normally results in safe RTE products (Table 8 and Supporting Information 2). In particular, *Campylobacter* spp., *L. monocytogenes*, *Salmonella* spp., and *Staphylococcus aureus* were not detected in RTE raw‐ripened wild boar loins with added curing salts and apple vinegar, though *S. aureus* was isolated from the control sample at Days zero (0) and 14 postproductions (Łepecka et al. 2023). The presence of *Mycobacterium* spp. in naturally infected game meat and organs (African buffalo and greater kudu) was also eliminated in the produced cured‐dried biltong, though obtained colonies on agar media originating from the untreated pooled control muscle tissue sample were identified as *M. bovis* by PCR (Van der Merwe and Michel 2010). Likewise, no mycobacteria were detected in sausages from wild boar meat (*Sus scrofa*) artificially contaminated with *M. bovis* that were subjected to a ripening/aging process for over 5 weeks, characteristic of cured meat, though viable (culturable) *M. bovis* was detected in intermediate products until Day 23 (Clausi et al. 2021). However, *L. monocytogenes* was present in 38% of RTE cured game meat sausages analyzed in four processing plants in Italy, but still under the applicable legal limit of 100 cfu/g and consistently under the method's limit of quantification (10 colony forming units/g; Commission Regulation (EC) No 2073/2005 of November 15, 2005 on Microbiological Criteria for Foodstuffs 2020; Lucchini et al. 2014). In addition, Díaz‐Sánchez et al. (2012) detected specific serotypes and encoding genes of STEC in 9.5% of wild boar and 2.7% of red deer RTE products (red cured sausages, cured sausages, and dry‐cured meats).

**TABLE 8:** Studies on fermented and cured game meat products.

Products (samples)	Country	Type of processing	Obtained result	References
Salame di daino (dry‐cured sausage; *Dama dama* and pork meat; *n* = 90)	Italy	Fermentation and ripening conditions: 6°C–20°C and RH 65%–90% (h 0–24); 15°C–16°C and RH 85%–90% (Days 1–21).	Absence of *Escherichia coli*; *Listeria* spp. and *Salmonella* spp. after 27 days of ripening	Cenci Goga et al. 2015
Wild boar sausages (*n* = 18)	Italy	Sausage stored at room temperature (10°C–18°C) in a controlled environment for 37–43 days	*Mycobacterium bovis* detected until Day 23 but not in the end products (Day 37–43)	Clausi et al. 2021
RTE game meat products from wild boar (WB) and red deer (RD): red cured sausages (WB *n* = 14; RD *n* = 9), cured sausages (WB *n* = 14; RD *n* = 10) and dry‐cured meats (WB *n* = 9; RD *n* = 2).	Spain	No available information	Shiga toxin‐producing *E. coli* (STEC) *stx* genes in 2/37 (5.4%) of the Wild boar RTE products and 4/21 (19%) of the Red deer RTE products	Diaz‐Sanchez et al. 2012
Homemade wild boar meat products (19 hams, 42 salami, and 22 raw/“Knackwurst” sausages)	Germany	Dry‐cured hams with and without smoking and with different ripening times Salame Fermentation: 25°C, RH: 88%–90%, 24 h Drying: 26°C, RH: 40%–60%, until Day 10 Fermented sausages: Drying: 26°C, RH: 40%–60%, 7 days	Effective inactivation of *Alaria alata* mesocercariae in all the products at the end of the processing time	González‐Fuentes et al. 2014
Japanese sika deer (*Cervus nippon centralis*) Cured meat (*n* = 8)	Japan	Meat soaked or rubbed with 2.0 or 6.0% NaCl and/or nitrite‐ enriched curing salt and stored at 4°C for up to 7 days.	More than 2.0% salt and NCS were effective in reducing the viability of *Sarcocystis* spp.	Honda et al. 2018
Raw‐aged wild boar loin	Poland	Product cured with salt and sodium nitrate and with or without addition of apple vinegar (4%). Maturing process = 15°C–17°C and humidity of 75%–80% for 2–3 days. Cold smoking at 20°C–25°C for 1–1.5 h. Maturation continues for 4 weeks with the assumed initial parameters of the process and low air movement. Products were vacuum‐packed and placed in a cooling room (4°C).	Coagulase‐positive staphylococci were found in samples without apple vinegar even after Day 28. *Staphylococcus aureus*, *Salmonella* spp., *Listeria* spp. and *Campylobacter* spp. were not found in any of the samples.	Łepecka et al. 2023
Samples of game meat from different specie including cured sausages (*n* = 59)	Italy	No available information	*L. monocytogenes* was present in 38% of the ready‐for‐sale cured sausages but under the legal limit of 100 cfu/g	Lucchini et al. 2014
Wild boar meat sausages and Deer meat sausages (1:1 ratio with pork; *n* = 105)	Croatia	Products with no starter cultures or nitrites. RH 52%–92%; −3°C to 15°C; cold intermittent smoking first 2 weeks and ripened for 20–40 days.	*Bacillus cereus* group < 1.0–5.7 log cfu/g; *Listeria monocytogenes* not detected in the end products (but detected during fermentation 2 log cfu/g); *Salmonella* spp. not detected; *S. aureus* < 1.0 log cfu/g (sporadically presented up to 20 days)	Maksimovic et al. 2018
Venison jerky (^a^)	United States	No available information (Outbreak)	*Clostridium botulinum*, type F toxin detected in the product	Midura et al. 1972
Wild boar sausages/salami (*n* = 63)	Italy	No available information	4 of 63 (6.3%) wild boar salamis—made without liver—were RT‐qPCR positive for Hepatitis E virus	Montone et al. 2019
Wild boar meat ripened sausages (mixed with pork; *n* = 72)	Croatia	Sausage with different starter culture. Fermentation/Ripening: 40 days, four smoking treatments	*S. aureus* and *Salmonella* spp. not detected in any treatment at any time. *L. monocytogenes*: Present in all treatments except one produced adding native Lactococcus (absent at Day 7 and in final product).	Mrkonjic Fuka et al. 2021
Bresaola from deer (*n* = 8) and wild boar (*n* = 8)	Italy	Salted at 2°C–4°C and left for 5–7 days at 3°C–4°C. Cured: Initial temperature 22°C and RH 90%, after 24 h temperature 18°C and RH 80%. Two drying stages: 17°C and RH 80% for 15–30 days, then 14°C and RH 72% for 60–120 days.	No *Salmonella, L. monocytogenes*, Clostridia or Pathogenic staphylococci were detected	Paleari et al. 2003
Wild boar sausages and dried muscle	Serbia	No available information	Presence of 0.18 lpg (*Τrichinella britovi* larva per gram) in dried muscle and 0.87 lpg in the sausages	Pavic et al. 2020
Dried wild boar ham	France	No available information (outbreak)	Presence of 8.32 lpg *Τrichinella britovi*	Peju et al. 2023
Traditional salami made from roe‐deer (*Capreolus capreolus*) meat and pork	Italy	Stuffed meat mix (fresh salami) was dried 10 days at 22°C and 62% relative humidity (RH) for 48 h; 19°C and 66% RH for 76 h, followed by a 1°C temperature reduction and 1% increase in the RH each day, so as to reach 15°C and 72% RH within 10 days). The products were	No *L. monocytogenes* and *Salmonella* spp. were detected in any sample at any time point.	Ranucci et al. 2019
		ripened in controlled seasoning rooms at 13°C and 75% RH for 60 days.		
Traditional salami made from wild boar meat and pork	Italy	Stuffed meat mix (fresh salami) was dried 10 days at 22°C and 62% RH for 48 h, 19°C and 66% RH for 76 h followed by 1°C T reduction and 1% RH increase every 24 h until reaching 15°C and 72% RH. Afterwards, the salami were ripened in controlled seasoning rooms at 13°C and 75% RH for 60 days	No *L. monocytogenes* and *Salmonella* spp. were detected in the final products	Roila et al. 2021
Salami from springbok (*Antidorcas marsupialis*), gemsbok (*Oryx gazella*), kudu (*Tragelaphus strepsiceros*) and zebra (*Equus burchelli*)	Namibia	Fermentation for 12 h at 18°C–22°C and RH 92%. Cold smoking with oak wood chips for 36 h at 22°C–24°C and RH 92%. Ripening: for a further 21 days at 12°C–16°C and RH 75%. Vacuum packaging and storing at 4°C.	S*taphylococcus aureus* below the detection limit in all the products	van Schalkwyk et al. 2011
Dried‐cured wild boar sausages (*n* = 2)	Italy	No available information (outbreak)	Presence of 1.5 and 1.7 lpg *Τrichinella britovi*	Turiac et al. 2017
Biltong from African buffalo (*Syncerus cafer*) and greater kudu (*Tragelaphus stepsiceros*)	South Africa	No available information	No *Mycobacterium* spp. were detected	Van der Merwe and Michel 2010

Review papers excluded.

^a^ Number of samples not available.

Fermented game meat products produced with either autochthonous or starter cultures of lactic acid bacteria (LAB) also exhibit acceptable overall food safety attributes with respect to bacterial foodborne pathogens (Table 8). Even in studies where pathogenic bacteria were originally present in raw meat mixtures and intermediate products, the fermentation process eliminated their presence in the final game meat products. For example, *L. monocytogenes*, *Salmonella* spp., and/or *S. aureus* were present at the initial stages of ripening but subsequently absent in the RTE venison (*Dama dama*) nitrite‐free dry‐cured sausages as well as in wild boar and deer (*Cervus elaphus*) starter culture‐free meat sausages (Paleari et al. 2003; Cenci Goga et al. 2012; Maksimovic et al. 2018; Mrkonjic Fuka et al. 2021). Also, *Salmonella enterica* serovar Typhimurium was detected in the meat batter of one batch and in 7‐day‐old salami samples produced from wild boar but was not detected from Day 14 and beyond, and *Salmonella* serovar Rissen was detected in the meat batter of another batch but not in corresponding salami samples tested on Day 7 and beyond (Roila et al. 2021). Nonetheless, other studies have reported the isolation and enumeration of *Bacillus cereus* group bacteria in spontaneously fermented game meat sausages (wild boar or deer meat with pork in 1:1 ratio; Maksimovic et al. 2018) and of sulfite‐reducing bacteria in salami samples (Ranucci et al. 2019). A detailed *E. coli* O157:H7 outbreak linked to consumption of jerky prepared from a black‐tailed deer in Oregon, USA is reported by Keene et al. (1997). The outbreak affected members of a household as well as their close family and friends. In November 1995, *E. coli* O157:H7 was cultured from a 3‐year‐old boy who presented with bloody diarrhea. Two days before the onset of symptoms, the family had made jerky from a deer that had been hunted the previous week. Ultimately, six confirmed and five presumptive cases were identified, with affected individuals ranging in age from 9 months to 54 years. The authors concluded that deer can be colonized by *E. coli* O157:H7 and can serve as a source of human infection.

Another article describes laboratory work performed in connection with the outbreak from 1966 in California, the only recorded case in US history of *Clostridium botulinum* toxin type F poisoning, originating from venison jerky consumed by humans. Researchers examined samples of the jerky using standard tests for *C. botulinum*, including selective culture methods and biological assays confirming the presence of type F botulinum toxin. The presence of the toxin in the jerky was confirmed, but no toxin or *C. botulinum* organisms could be detected in any of the ingredients (seasoned salt, black pepper, curing sugar, and Bar‐B‐Q liquid smoke) or in enrichment cultures of these products. The investigation highlights the need for caution in the preparation and consumption of meat products, especially dried game meat (Midura et al. 1972).

Regarding parasitic hazards, wild boar (*Sus scrofa*) hunting is widespread in many European countries, and meat from these animals is often used to produce traditional products such as sausages and hams. Human trichinellosis outbreaks continue to be reported regularly across the continent, typically following the consumption of uncontrolled wild boar meat products that have not undergone the mandatory *Trichinella* testing required under EU legislation (EU Reg. 2015/1375, Noeckler et al. 2019). Three recent studies reported *T. britovi* in wild boar meat from France (Peju et al. 2023), Italy (Turiac et al. 2017), and Serbia (Pavic et al. 2020), with confirmed human trichinellosis outbreaks linked to the consumption of raw or dried homemade sausages and ham. Infected meat contained up to 17 larvae/g, and diagnosis was confirmed via artificial digestion of the meat and PCR. One study highlights the need to raise awareness among hunters about the risks associated with consuming raw game meat, and that freezing meat does not guarantee the inactivation of larvae (Peju et al. 2023).

Other parasite species can also be found in wild boar meat in Europe. In 2015, González‐Fuentes et al. studied *A. alata* mesocercariae in wild boar meat used to produce traditional German raw cured products. Viable parasites were detected in raw sausages 24 h postpreparation but were completely absent in final products after standard processes, including curing with nitrite salt, fermentation, cold smoking, and drying for 7–22 days, depending on the product type. Another study examined *Sarcocystis* spp. in Japanese sika deer meat and all 20 samples were positive. Viability was lost within 1 day when cured with ≥2.0% salt with or without nitrite. In contrast, the parasites survived for at least 7 days at refrigeration temperatures (0°C–4°C) and under acidic conditions (pH 3.0 – 5.0), indicating limited effect of cold storage or acidification alone (Honda et al. 2018).

HEV has emerged as a notable foodborne hazard, with pork and game meat implicated in most locally acquired cases. Montone et al. (2019) surveyed 162 cured or raw meat products sold in Campania (Italy) and detected HEV RNA in four out of 63 home‐made wild‐boar sausages (6.3%), while none of the 99 pork samples were confirmed positive. Viral loads in the sausages were low (≈2–3 log10 copies/g), yet these products are often eaten uncooked, so viability and therefore infection risk, remains uncertain. Phylogenetic analysis identified all sequences as genotype 3 (clade 3abchij) and closely related to strains previously found in local wild‐boar livers, underscoring spill‐over from wildlife reservoirs. Although limited in prevalence, this study highlights wild‐boar meat as a credible vehicle for zoonotic HEV and supports its inclusion in quantitative risk assessments of viruses in game meat.

### 4.4 Other Processing Methods

Processes including marinating, antimicrobial use, smoking that are applied to game meat are reviewed in this section.

In recent years, the demand for natural preservatives in the preservation of meat and meat products has increased (Efenberger‐Szmechtyk et al. 2021). In this context, there are studies investigating the effects of polyphenols obtained from plants on pathogens and spoilage microorganisms (Rodriguez Vaquero et al. 2013; Zhao et al. 2019; Kalogianni et al. 2020; Roila et al. 2022a). However, there have been a limited number of studies on the antimicrobial effects of polyphenols in game meats (Altissimi et al. 2024; Roila et al. 2022b; Kačániová et al. 2024a, 2024b). In a study, Altissimi et al. (2024) found that counts such as aerobic colony count, *Enterobacteriaceae*, *Pseudomonas* spp. and LAB counts were significantly reduced after 7 and 14 days of storage under vacuum‐packed conditions at 3°C in game meats immersed in 10% polyphenol solutions obtained from olive mill wastewater, which mostly contain hydroxytyrosol and tyrosol.

In another study, the effect of 2% lactic acid and aromatic vinegar solutions on microbial load reduction on the surfaces of wild boar carcasses was investigated. Both lactic acid and aromatic vinegar have been found to provide a reduction in the number of aerobic colonies, the number of *Staphylococcus* spp. and the number of *Lactobacillus* spp. In particular, it was noted in the study that the application of lactic acid solution was highly effective in reducing the microbial load on wild boar carcasses (Roila et al. 2022b).

In their study, Kačániová et al. (2024a) investigated the antimicrobial effect of vacuum packaging in red venison with the application of *Piper nigrum* essential oil (PNEO). As a result of the study, it was shown that the total viable count, coliform bacteria and *L. monocytogenes* counts decreased with the temperature and processing time of the sous vide method. The lowest counts in the microorganism groups were observed in samples treated with 1% PNEO. The analysis revealed that PNEO, in combination with the sous vide method, effectively reduced *L. monocytogenes* counts and extended the shelf ‐life of red deer meat.

In a similar study, researchers investigated the effect of *Eugenia caryophyllus* essential oil on *Salmonella enterica* in red deer meat. As a result of the analyses, it was determined that *Eugenia caryophyllus* essential oil showed strong antibacterial activity against *S. enterica*. In addition, the antibiofilm activity of *Eugenia caryophyllus* essential oil was also detected in that study (Kačániová et al. 2024b).

In Katanga, the southern province of the Democratic Republic of the Congo, it was reported that smoked game meat is often consumed by rural populations living around forested areas. In these regions, smoked game meat is highly appreciated by consumers for its organoleptic properties such as taste, smell and color. Smoking is a preservation method that aims to extend the shelf‐ life of meat and improve its organoleptic properties. This method has been developed in the region, especially for the transportation of meat from areas far from settlements without spoiling. Thus, game meat obtained in rural areas can be transported to vendors in urban centers in the form of dried or smoked meat (Mpalang et al. 2013). In a study investigating the bacteriological quality of smoked game meat in Lubumbashi, 182 samples of smoked game meat belonging to three species: *Syncerus caffer* (African buffalo; *n* = 63), *Phacochoerus aethiopicus* (desert warthog; *n* = 60) and *Sylvicapra grimmia* (common duiker; *n* = 59) sold in retail outlets were analyzed. *E. coli* was isolated from 81.3% of the samples, indicating that significant fecal contamination occurred in smoked game meat. In the study, Shiga toxigenic *E. coli* (STEC), *Salmonella* spp., *Campylobacter jejuni* and *Campylobacter coli* were detected in 0.0%, 4.3%, 3.8%, and 14.2%, respectively. These data showed that smoking in Lubumbashi was not sufficient to ensure the safety of game meat (Mpalang et al. 2013).

Among the innovative methods which could be applied for game meat safety based on the other information, bacteriocins effect can be allotted (Franz et al. 2007; Gillor et al. 2008; Ness et al. 2014). Bacteriocins represent antimicrobial active substances of proteinaceous character with inhibitory effect against more or less relative bacteria which are produced by various bacterial strain species (Ness et al. 2014); often involving also those bacterial strains with probiotic character (Franz et al. 2007). Recently, bacteriocins were allotted in the group of postbiotics, namely, preparations of inanimate microorganisms and/or their components that confers a health (Salminen et al. 2021), meaning different biological components with beneficial influence on host niche and/or organism. They are more often indicated as components providing new horizons in microbial biotherapy, and /or preventing and functional foods (Nataraj et al. 2020). No studies on the use of bacteriocins on game meat products are present in the databases but Nisin was successfully used in prevention against *Salmonella* spp. and *S. aureus* colonies in fresh pork meat. Antilisterial effect of nisin was demonstrated in Gombasek sausage (up to difference 4.4 log cycle) and Púchov salami with the almost same antimicrobial effect without negative influence on sausage pH value (Lauková et al. 2003; Lauková and Turek 2012).

Considering other processing technologies, the effects of irradiating wild boar muscle experimentally infected with *T. spiralis* and *T. pseudospiralis* is reported. A dose of 0.32–0.41 kGy of Cobalt 60 was used on the muscles and the absence of viable parasitic larvae in the meat was highlighted already after 24 h from the treatment (Ercole et al. 2021). Nonetheless, meat irradiation is not allowed in several countries worldwide (European Commission 2021).

## 5 Discussion

The results of this systematic review reveal that, although there is an increasing demand in game meat as it is perceived as organic and more natural, product safety is not sufficiently controlled. Variable hunting hygiene, insufficient processing standards and a lack of consumer knowledge regarding preparation methods lead to a high biological risk in game meat. Pathogens such as *L. monocytogenes*, *Salmonella* spp., *E. coli* (including STEC), *Trichinella* spp., and zoonotic viruses like HEV are still being identified in game meat and game meat products (Cook et al. 2000; Pozio 2007; Díaz‐Sánchez et al. 2012, 2013; Montone et al. 2019; Fredriksson‐Ahomaa et al. 2022).

Cold‐treatment and freezing are standard methods for preserving game meat but their effectiveness in controlling bacteria and parasites is limited and varies by species. Although several pathogen species are eliminated with freeze‐treatment (*T. spiralis, T. gondii*; Kotula et al. 1991; Kijlstra and Jongert 2008), freeze‐resistant pathogens such as *T. nativa* and *T. britovi* and low‐temperature growing bacteria pose significant public health risks, even after extended freezing periods, necessitating caution among hunters, meat processors, and consumers (Gamble et al. 2000; Pozio 2019). Among several foodborne pathogenic bacteria, the presence in refrigerated game meat of well‐established foodborne pathogens, such as *L. monocytogenes*, *Yersinia* spp., *Salmonella* spp., pathogenic *E. coli*, and *Campylobacter* spp. poses a significant risk (Hedman et al. 2020). These pathogens are closely linked to hygiene standards and are capable of surviving, and in some cases multiplying, at low temperatures. Consequently, their presence in game animals and the potential for contamination of meat and proliferation during cold storage may increase the associated health risks—an area that remains under‐researched.

Curing of game meat generally results in safe RTE products, regarding foodborne bacterial pathogens (Table 7), but tends to be insufficient for eliminating parasites such as *Trichinella* spp. or *A. alata* (González‐Fuentes et al. 2015; Pavic et al. 2020; Peju et al. 2023; Turiac et al. 2017). Historically, cured game meats have been implicated in foodborne outbreaks; however, these incidents occurred in the past and were likely associated with reduced vigilance and less stringent food safety practices at the time. Today, commercially produced cured meat products are generally considered safe, as modern preservation techniques help ensure their microbiological stability. Nevertheless, caution remains warranted regarding the potential survival of STEC and *L. monocytogenes*, which appear capable of surviving certain curing processes. Only one study was found on the effect of curing on viruses in game meat and although limited in prevalence, this study highlights wild boar meat as a credible vehicle for zoonotic HEV and supports its inclusion in quantitative risk assessments of viruses in game meat (Montone et al. 2019).

Only six studies were found on other processes, including marinating, antimicrobial use, smoking, and drying. Natural substances, such as polyphenols derived from olive oil or plant essential oils with potent antimicrobial properties are expected, in combination with the appropriate packaging, to provide organoleptically sound and microbiologically stable products, as shown in meat products in general (Kalogianni et al. 2020; Da Silva et al. 2021). However, the rather small number of published studies and the quite variable initial microbial flora of game meat, do not give a clear picture of their importance. Concerning other traditionally used preservation methods, such as smoking and drying, again the number of studies is rather limited; it is highly unlikely that these methods can ensure game meat safety, especially since dried products differ considerably concerning parameters that permit microbial proliferation (Mediani et al. 2022). Their contribution to a hurdle technology system should not be overlooked, as they are certainly an integral part of the production process for certain high added value game meat products.

Thermal processing remains one of the most traditional and straightforward techniques for inactivating foodborne pathogens. While specific studies on heat treatment in game meat are relatively sparse, existing evidence strongly supports that high‐temperature thermal processing—whether sous vide, conventional cooking, or heat drying—is effective in inactivating a broad spectrum of bacterial pathogens and parasites. Given the variability in meat types and processing methods, proper heat treatment remains essential for ensuring the microbiological safety of game meat products.

In general, there is a lack of standardized processing and hygiene protocols specifically for game meat. There are currently no established microbial contamination thresholds specifically for game meat, therefore, guidelines for livestock are usually applied (Needham 2023). Among the bacterial indicators commonly reported in meat and meat products, aerobic viable counts, as well as counts of *E. coli* and Enterobacterales, were frequently reported, with considerable variation in average values (e.g., Tables 5 and 6). In several cases, these indicators exceeded the microbial criteria established by EU legislation (Commission Regulation (EC) No 2073/2005 of November 15, 2005 on Microbiological Criteria for Foodstuffs 2020) and any subsequent amendment) for carcass surfaces, whereas in other instances, the mean values were significantly lower (Peruzy et al. 2022). This considerable variance in the initial microbial flora may inadequately reflect the risk profiles of game meat, making the estimation of the efficiency of preservation techniques quite difficult. While freezing, chilling, and cooking remain cornerstones of microbial control, their effectiveness is highly dependent on precise parameters—many of which were incompletely reported in the literature surveyed. For instance, only 17 out of 64 reviewed records included a full description of the technological process applied, significantly limiting the other studies’ utility for risk assessment or regulatory refinement. The lack of a full description of the process, makes it difficult to attribute microbial reductions to specific technological interventions. Additionally, most records involved mixed meat formulations (e.g., game–pork blends), complicating risk attribution.

Despite extensive focus on bacterial hazards (47 studies), limited data were available on viruses (two studies), parasites (18 studies) and transmissible spongiform encephalopathy (TSE) such as chronic wasting disease (CWD) in game meat. Only two studies specifically addressed zoonotic viruses, even though HEV has been repeatedly detected in raw or minimally processed wild boar sausages. Similarly, many parasitic threats (e.g., *A. alata*, *Sarcocystis* spp., and liver flukes) remain under‐recognized and poorly documented, particularly in cured or fermented products. Their survival under common processing conditions warrants further investigation.

Based on the previous experiences with bacteriocins (postbiotic) use in food products involving meat, it is a chance for their use also for game meat treatment. There is a lack and/or no information using this treatment associated with game meat. So, it is also an open area for continuing research in this field.

Given the limited availability of indexed scientific literature on certain aspects of game meat processing, only two databases (PubMed and Google Scholar) were searched for the present review. Although this combination has been shown to retrieve a substantial proportion of literature (Bramer et al. 2013; Teo et al. 2020), restricting the search to a limited number of sources may have resulted in missing relevant studies. Future updates of the review should therefore broaden the search to additional databases to enhance completeness and minimize potential selection bias.

## 6 Conclusions

This systematic review clearly highlights the significant microbiological risks associated with game meat, despite its increasing popularity as a natural and organic food source. The lack of standardized hygiene practices during hunting and processing, combined with insufficient consumer awareness, allows foodborne pathogens to persist in game meat products. While preservation techniques like refrigeration and freezing are commonly applied, their efficacy is inconsistent. Freezing may inactivate certain parasites, but it is mostly ineffective against freeze‐resistant species such as bacteria and viruses. Furthermore, although curing has demonstrated some microbiological safety in contemporary ready‐to‐eat products, it fails to eliminate specific parasites and resistant bacteria. Alarmingly, only a single study has investigated viral inactivation during curing, revealing a significant knowledge gap, particularly regarding HEV. Thermal processing stands out as the most effective method for pathogen reduction; however, there is a notable lack of studies focusing specifically on its application to game meat. Alternative methods, such as marinating, smoking, drying, and the use of natural antimicrobials, have yet to receive thorough exploration and warrant further investigation. A critical limitation across the reviewed literature is the inadequate reporting of processing parameters, which hinders accurate assessment of intervention efficacy. Additionally, the frequent use of mixed meat formulations complicates risk attribution. The overwhelming focus on bacterial hazards, with far fewer studies addressing viruses and parasites, indicates a glaring imbalance in current research efforts.

In conclusion, ensuring the microbiological safety of game meat demands the establishment of stringent hygiene protocols, targeted processing guidelines, regular inspections, and robust consumer education. Future research must decisively address the identified gaps, particularly concerning viral, parasitic, and chemical residual risks, to support the creation of regulatory standards specifically designed for the unique characteristics of game meat production and consumption. Regulatory guidelines should acknowledge the limitations of the different processes and standardize processing protocols regarding the handling of game meat. Hunters, small‐scale producers, and consumers must be better educated about biological risks, residues, safe processing parameters, and effective cooking practices.

## Author Contributions

**Naim Deniz Ayaz**: writing – original draft, writing – review and editing, data curation, conceptualization. **Ali Aydin**: writing – original draft, writing – review and editing, data curation, conceptualization. **Ewa Bilska‐Zając**: writing – original draft, data curation, writing – review and editing, conceptualization. **Raffaella Branciari**: data curation, writing – original draft, writing – review and editing, conceptualization. **Gunita Deksne**: writing – review and editing, data curation, writing – original draft, conceptualization. **Vangelis Economou**: writing – original draft, writing – review and editing, data curation, conceptualization. **Bożena Futoma‐Kołoch**: writing – original draft, data curation, writing – review and editing, conceptualization. **Robert Głogowski**: writing – original draft, data curation, writing – review and editing, conceptualization. **Eduarda Gomes Neves**: writing – original draft, writing – review and editing, data curation, conceptualization. **Famke Jansen**: writing – original draft, writing – review and editing, data curation, conceptualization. **Weronika Korpysa‐Dzirba**: writing – original draft, conceptualization, data curation, writing – review and editing. **Andrea Lauková**: writing – original draft, conceptualization, data curation, writing – review and editing. **Thomai Lazou**: writing – original draft, conceptualization, data curation, writing – review and editing. **Guðný Rut Pálsdóttir**: writing – original draft, conceptualization, data curation, writing – review and editing. **Maria Francesca Peruzy**: writing – original draft, conceptualization, data curation, writing – review and editing. **Petras Prakas**: writing – original draft, conceptualization, data curation, writing – review and editing. **David Ranucci**: data curation, writing – original draft, writing – review and editing, conceptualization. **Rossana Roila**: data curation, conceptualization, writing – original draft, writing – review and editing. **Mirosław Różycki**: writing – original draft, conceptualization, data curation, writing – review and editing. **Selene Rubiola**: writing – original draft, conceptualization, data curation, writing – review and editing. **Ioannis Sakaridis**: writing – original draft, conceptualization, data curation, writing – review and editing. **Madalena Vieira‐Pinto**: writing – review and editing, conceptualization, writing – original draft, data curation.

## Conflicts of Interest

The authors declare no conflicts of interest.

## Supporting information




**Supporting Information**: crf370420‐sup‐0001‐SuppMat.docx

**Supporting Information**: crf370420‐sup‐0002‐SuppMat.docx

## Data Availability

All records consulted for the systematic review are available at https://doi.org/10.5281/zenodo.18387147 ensuring transparency and reproducibility.
