# Supplementary material for: The Effectiveness of Postharvest Processing on Microbiological Safety of Game Meat—A Systematic Review
Source: Compr Rev Food Sci Food Saf. 2026 Feb 28;25(2):e70420. doi: 10.1111/1541-4337.70420 (PMC12949643; doi:10.1111/1541-4337.70420)
Supplement: Supplementary file 1 — Supporting Information: crf370420‐sup‐0001‐SuppMat.docx [file CRF3-25-e70420-s001.docx]

**SM1: Supplementary Material 1**

**Glossary**

Game meat refers to the flesh of wild animals hunted for food, distinct from domesticated meats like beef, lamb/mutton, pork, chicken or turkey. It encompasses a variety of species, such as deer, wild boar, elk, rabbits, and birds like pheasants, ducks, and partridge. Game meat is highly appreciated by some for its unique flavor and lean texture reflecting the animal's wild/natural diet and active lifestyle. Although game meat consumption may vary depending on cultural and regional differences, it has a low amount in total meat consumption and is generally not reported as a separate category in official statistics.

Terminology surrounding game meat has evolved, often varying among hunters, consumers, media, and scientists. Discrepancies in definitions can cause misunderstandings about production, safety, and consumption. A standardized vocabulary is essential for clarity and consistency in the field. Terminology related to game meat generally covers the species of animals hunted, equipment used, and the specific conditions of hunting.

Generally, “Meat” refers skeletal muscle and related tissues from mammals, birds, reptiles, and amphibians. Red/white meat is a traditional classification based on species, color, and fat content. Red meat typically includes beef, pork, and lamb, while white meat refers to chicken and turkey breast.

Due to the animal species game meat can be categorized into three main groups:

- Winged game: Birds like pheasants, partridge, quail, ducks, and turkeys, often classified as "upland birds" or "waterfowl."
- Ground/small game: Smaller animals like rabbits, hares, and beavers, often used for stews or sausages.
- Big game: Larger animals such as deer, elk, and wild boar, valued for their rich flavor and typically aged or marinated for preparation.

Hunters also contribute a rich lexicon of traditional terms, reflecting cultural practices. For example, “virgin meat” describes tender meat from young animals, while “trophy carcass” refers to animals prized for their antlers or horns. This terminology highlights both practical knowledge and cultural heritage, enriching the understanding of game meat.

There are also terms related to the hunting process. These are;

• Harvesting: Hunting and using game animals under control.

• Field dressing: Removing the internal organs of a game animal after hunting.

• Skinning: Removing the animal's hide.

• Maturation (Aging): Waiting for a controlled period of time to increase the flavor of game meat.

If the subject is discussed in more detail. Meat processing refers the transformation of raw carcass meat into consumer-ready products through physical or biochemical methods, ranging from minimal handling to extensive processing. This covers various stages and terms such as;

- Aging (Ripening): A process in the production of cured and fermented game meats, contributing to flavor development, texture enhancement, and preservation. This process involves the aging of meat under controlled conditions, allowing enzymatic and microbial activities to transform the meat's characteristics. Ripening is commonly used in products like salami, prosciutto, and other cured meats. Some hunters also use terms like "field aging" when discussing the initial resting of the carcass in the field before transportation, emphasizing proper temperature management to avoid spoilage. The duration of aging varies depending on the species, age of the animal, and the desired flavor profile. Terms such as "young aging" may be used for quick processes applied to tender venison, while "deep aging" refers to extended periods that develop stronger, gamier flavors. In general, it’s a process of storing meat at temperatures between 0-4°C with a humidity level of 85-90% for 2-28 days.
- Ante mortem inspection: A visual examination of the animal immediately before kill to ensure it is healthy and suitable for consumption.
- Aspic: A dish of cooked game meat suspended in clarified, gelatinized stock, served cold as a savory appetizer.
- Barding: The wrapping of the game meat in a layer of fat (e.g., bacon or caul fat) before roasting or grilling. This protects the meat from direct heat and keeps it moist during cooking.
- Bleeding: Immediate exsanguination is critical. Carcasses should ideally lose 50-55% of their total blood volume within 10-15 minutes post-mortem. Delays can cause rapid bacterial growth and meat discoloration.
- Boning (Deboning): The removal of bones from meat, often done at a controlled temperature of 10-12°C (50-54°F) to make cuts easier to process. Commonly used for venison, wild boar, and small game.
- Brining: Soaking meat in a saltwater solution for several hours to improve juiciness and flavor before further processing, commonly used for wild birds or small game mammals.
- Butchering: Dividing the carcass into smaller, manageable cuts at ambient temperatures of 10-12°C, with duration depending on the size of the carcass. This process is used for larger game animals, such as deer, elk, and wild boar.
- Canning: Placing seasoned meat into sterilized jars or cans, then sealing and heating at 121°C for 75-90 minutes for sterility and extended shelf life.
- Caping: The careful removal of the animal's skin, especially around the head, for trophy purposes. This is done to preserve the hide intact for mounting.
- Carcass breakdown and aging: For dry aging, the meat is stored in a controlled room at temperatures between 0°C and 2°C, with humidity levels maintained between 80-85%. Aging periods range from 2 to 28 days depending on the species and desired flavor profile. Wet aging is done in vacuum-sealed bags and aged at 0°C to 4°C for up to 10 days.
- Cold chain management: The temperature should remain at or below 7°C throughout transport to the processing facility, following strict cold chain guidelines.
- Decontamination: reducing microbial load on meat surfaces using chemical washes, like lactic acid, or physical methods such as steam, fire applied directly after slaughter.
- Disinfection: The elimination or reduction of microorganisms on surfaces using chemical agents (e.g., chlorine) or UV light, with timing based on the chosen method.
- Dry-curing: Rubbing meat (e.g., wild boar ham or venison salami) with salt, sugar, and spices, and hanging it at 10-15°C with 70-80% humidity for weeks to months for flavor development and preservation.
- Evisceration: Removal of internal organs from the carcass immediately after slaughter, typically taking 10-20 minutes. Necessary for all game species, including deer, wild boar, and rabbits.
- Emulsification: A critical process in meat processing, particularly in the production of emulsified meat products such as sausages, pâtés, and terrines. It involves the stable blending of fat and water with lean meat proteins to create a uniform, smooth texture.
- Fermentation: Using beneficial bacteria to develop flavor and preserve meat at 20-30°C with 85-90% humidity for 1-3 days. Applied in the production of fermented sausages from wild boar and venison.
- Field dressing: Removal of internal organs in the field immediately after harvest to maintain meat quality, commonly practiced with deer, elk, and wild boar.
- Freezing: Preserves meat by lowering its temperature to-30°C to -18°C, inhibiting microbial and enzymatic activity. Optimal storage time ranges from 6-12 months.
- Forcemeat: A finely or coarsely ground mixture of meat, fat, and seasonings, often emulsified, used in sausages, pâtés, terrines, and stuffed dishes.
- Grinding: Breaking meat into smaller pieces or paste at temperatures of 10-12°C. Used for producing ground meat from venison, wild boar, and rabbits.
- Haunch processing: Separating the haunch, or upper hind leg, from the rest of the carcass. This cut is a favorite for roasting and is often handled with care to preserve the tenderest portions.
- Hunting: Hunters are typically required to follow local or international animal welfare standards, ensuring a clean, fast kill to avoid stress-induced meat degradation (PSE: pale, soft, exudative meat).
- Injection marination: For larger cuts, automated injector systems are used, injecting marinades at 10-15% of the total weight of the meat. This allows even penetration of flavors and accelerates the marination process.
- Post-mortem inspection: Ensuring meat safety and quality through visual and physical checks at various processing stages.
- Jointing: Breaking down the carcass at the joints to separate limbs and other sections, making the meat easier to handle, cook, or freeze. Used for large game animals and some birds.
- Larding and barding: Traditional techniques used to add fat to lean game meat to prevent drying out during cooking. Involves inserting strips of fat (often pork fat) into the meat using a larding needle.
- Long-curing: Curing meat with salt and spices, then aging it in a controlled environment at 10-15°C with around 80% humidity for several months, producing products like venison prosciutto and wild boar salami.
- Modified Atmosphere Packaging (MAP): MAP is often used for game meat to maintain freshness, using a gas mixture of 60-70% CO₂ and 30-40% N₂, with an oxygen content of less than 1%. This extends the shelf life by reducing aerobic bacterial growth.
- Marination: Soaking meat in spices, herbs, and liquids at 4°C for 2-24 hours to enhance flavor and tenderness.
- Marinate: Marination can last from 12 to 48 hours, depending on the thickness of the cuts. Common marinades have a pH range of 3.5 to 5.0 and include acidic components like vinegar (5-7% acetic acid) or wine, which aids in tenderization by breaking down protein fibers.
- Pâté production: For game pâtés, the meat is often finely minced and blended with 15-30% fat (typically pork or duck fat) to achieve a smooth texture. Cooking temperatures are kept at around 80°C to ensure pasteurization, and the final product is often vacuum-sealed.
- Pellicle formation: Allowing the meat surface to dry slightly before smoking, which forms a thin "skin" that helps smoke adhere to the meat for better flavor.
- Plucking: Feather removal from birds after scalding, taking 5-10 minutes.
- Rendering: Processing animal fat into lard or other products at 100-120°C for 1-2 hours.
- Retort pouch packaging: An alternative to traditional metal cans, retort pouches are made from flexible plastic and aluminum foil, which undergo heat treatment to sterilize the contents.
- Sausage production: A process of grinding and mixing game meat with fat, spices, and other ingredients, followed by stuffing the mixture into casings. This method allows for the production of flavorful, preserved, and versatile sausage products from wild game.
- Scalding: Immersing carcasses in hot water at 60-65°C for 2-5 minutes to aid in hair or feather removal.
- Scoring: Making shallow cuts on the surface of the meat to allow better penetration of marinades or seasoning, often used for tough cuts of game meat to improve flavor and tenderness.
- Skinning: Removal of the skin from the carcass.
- Stewing: A slow, moist-heat method where small, marinated game meat pieces are simmered at 80–90°C in a flavored liquid (e.g., stock or wine) for 1.5–3 hours to tenderize and develop rich flavors.
- Smoking: Exposing meat to smoke at 20-30°C for cold smoking or 60-85°C for hot smoking for 1-24 hours, which imparts flavor and aids preservation.
- Sous-vide: A precision cooking technique where game meat is vacuum-sealed and cooked at low, controlled temperatures for extended periods. This method enhances tenderness and retains moisture.
- Tallow rendering: Melting down fat from animals like wild boar to produce tallow, used for cooking or preservation. This fat has a distinct flavor that adds richness to certain game dishes.
- Tenderizing: A mechanical process (often using a tenderizer) or a chemical process (marinade or enzyme) to break down connective tissue in tough cuts, improving texture and palatability.
- Trimming: The removal of excess fat, silver skin, or other undesired portions of meat from the carcass or individual cuts. Proper trimming is essential for meat quality, preventing off-flavors and improving appearance.

Main terms related to hunting equipment are;

- Hunting rifle: Rifles used for hunting large game animals.
- Trapping: Methods used to catch game animals.
- Hound: A type of dog used in hunting.
