# Supplementary material for: The Effectiveness of Postharvest Processing on Microbiological Safety of Game Meat—A Systematic Review
Source: Compr Rev Food Sci Food Saf. 2026 Feb 28;25(2):e70420. doi: 10.1111/1541-4337.70420 (PMC12949643; doi:10.1111/1541-4337.70420)
Supplement: Supplementary file 2 — Supporting Information: crf370420‐sup‐0002‐SuppMat.docx [file CRF3-25-e70420-s002.docx]

**SM2: Supplementary material 2**

| **Type of product** | **Region, Country** | **Type of samples** | **Fermentation/Curing parameters** | **Product characteristics (e.g. pH, aw, weight)** | **Targeted pathogens** | **Detection method(s)** | **Results** | **Reference** |
| --- | --- | --- | --- | --- | --- | --- | --- | --- |
| Fermented | **Umbria, Italy** | *Salame di daino* (dry-cured sausage; *Dama dama* and pork meat) with and without (control) selected dairy-origin starter (SDS). n = 90: 10 salami productions (5 SDS, 5 controls), 3 sausages per group (control and starter), analysed at day 0 (immediately after stuffing), day 6 (end-fermentation), and day 27 (end-ripening process). | Frozen lean fallow deer meat (28%), pork shoulder and boneless belly (55%), and back fat (16%), mixed with NaCl (30 g/ kg), pepper (5g/ kg), garlic (2 g/Kg), white wine (2 ml/kg) and ascorbic acid (2g/ kg); no nitrites or nitrates were used; initial levels of 7 log cfu/g SDS cultures (cocci:bacilli = 2:1).  Fermentation and ripening conditions: 6 −20°C and RH 65 – 90% (hours 0 – 24); 15 − 16°C and RH 85 – 90% (days 1 – 21). | Each dry-cured sausage was 30 mm in diameter and 10 cm in length. Day 0: SDS (aw: 0.966, pH: 6.56); control (aw: 0.968, pH: 6.58)  Day 6: SDS (aw: 0.934, pH: 5.66); control (aw: 0.929, pH: 6.23)  Day 27: SDS (aw: 0.837, pH: 6.76); control (aw: 0.834, pH: 6.81) | Sulphite- reducing *Clostridium spp. C. botulinum Salmonella* spp*. Listeria* spp*. Listeria monocytogenes* Non-sorbitol fermenting *Escherichia coli S. aureus* | Classical culture-based methods -ISO standard microbiological methods. Samples yielding no typical colonies were recorded as negative and samples yielding at least one typical colony in the lowest dilution were recorded as positive, regardless of cfu. | Day 0: All SDS and control samples were negative for sulphite reducing *Clostridium* spp., *C. botulinum*, and non-sorbitol fermenting *Escherichia coli*; SDS samples positive for *Listeria* spp. (6/15) and *Salmonella* spp. (6/15); Control samples positive for *Listeria* spp. (8/15), *Salmonella* spp. (6/15), *S. aureus* (2/15).  Day 6: All SDS samples were negative for the targeted pathogens; Control samples positive for *Listeria* spp. (6/15), *Salmonella* spp. (6/15), *S. aureus* (2/15). Day 27: All SDS and control samples were negative for the targeted pathogens. | **Cenci Goga et al., 2012** |
| Cured | **Italy** | One sausage batch with wild boar meat (*Sus scrofa*) artificially contaminated with 2 g of wild boar pharyngeal lymph nodes with active tuberculous lesions that were previously tested positive for the presence of *M. bovis* by bacteriological examination.  Two sausage batches with wild boar (*Sus scrofa*) meat artificially contaminated with one *M. bovis* field strain at two different concentrations (5 log CFU/g and 3 log CFU/g). | Minced wild boar meat seasoned with salt and ground pepper. The sausage samples were stored at room temperature (10–18 ⁰C) in a controlled environment for 37–43 days and underwent a ripening/aging process, characteristic of cured meat. | Sausages of 100gr | *Mycobacterium bovis* | Classical culture-based methods and PCR were used to test the sausage samples every 7–10 days until the end of the ripening/aging process (37–43 days). | Viable (culturable) *M. bovis* was detected until day 23 in sausage samples post artificial contamination but not in the end products (37–43 days). | **Clausi et al., 2021** |
| Cured | **Castilla-LaMancha, Spain** | RTE game meat products from wild boar (WB) and red deer (RD): red cured sausages (WB n=14; RD n=9), cured sausages (WB n=14; RD n=10) and dry cured meats (WB n=9; RD n=2). | NA | NA | Shiga toxin-producing *E.coli* (STEC) | Classical culture-based methods for non-O157 STEC ISO standard microbiological method for *E. coli* O157:H7 PCR: STEC isolates were tested for *stx1* and *stx2* genes (three *stx1* and seven *stx2* subtypes) and *eae* and *ehxA* genes Pulsed-field gel electrophoresis (PFGE) among STEC isolates of the same serotype | Detected *stx* genes  Wild boar RTE products 2/37 (5.4 %) Red deer RTE products 4/21 (19 %)   STEC isolation and subtyping:  Wild boar RTE products 1/37 (2.7 %)  Serotype O11:H5 (*stx1 -, stx2 +, stx subtype 2a, eae -, ehxA +)*  Red deer RTE products 2/21 (9.5 %) Serotype O41/O179:H31 (*stx1 -, stx2 +, stx subtype 2g, eae -, ehxA +*) Serotype O166:H28 (*stx1 +, stx2 +, stx subtype 1c and 2b, eae -, ehxA +)* | **Diaz-Sanchez et al., 2012** |
| Fermented - Cured | **Brandenburg, Berlin, Saxony and Saxony-Anhalt, Germany** | Carcasses of wild boars (n = 18), highly infested with *Alaria alata* mesocercariae (arithmetic mean 80 DMS/100 g tissue), were used for the production of a total of 83 homemade meat products (19 hams, 42 salami and 22 raw/“Knackwurst” sausages). | RAW HAMS Wild boar hams for dry cured (group 1, n= 7) and cold smoking (group 2, n=8) products were spiked with additional 20 vital *A. alata* mesocercariae in order to increase the infestation rate, whereas hams of group 3 were not.  Curing: Each ham was submerged for 13 days at room temperature (18–20 °C) in stainless steel buckets containing 40 g nitrite curing salt. Curing mixture: salt, sodium hexacyanoferrate (II) E535 and 0.4–0.5% sodium nitrite.  Dry cured hams (group 1, n= 7): Curing Equalization/  washing/drying (26°C) ─ End of process (day 22).  Dry cured with cold smoking hams (group 2, n=8): Curing Equalization/  washing/drying ─ Cold smoking for 24 h (26°C) ─ Drying (26°C) ─ End of process (day 16).  Dry cured with cold smoking hams (group 3, n=8): Curing Equalization/ washing/drying ─ Cold smoking for 24 h (26°C) ─ Drying (26°C) ─ End of process (day 13).  SALAMIS *A. alata* mesocercariae positive meat, fat tissue, nitrite curing salt, starter culture (*Lactobacillus* *sakei* and *Staphylococcus carnosus*) Stuffing: Before clipping the 40 mm diameter casings, 20 additional vital *A. alata* mesocercariae were spiked to the filling (batch 1) or not (batch 2) Fermentation: 25 °C, RH: 88-90%, 24 h Drying: 26 °C, RH: 40-60%, until day 10  FERMENTED RAW-TYPE SAUSAGES  *A. alata* mesocercariae positive meat, fat tissue, nitrite curing salt, Stuffing: Before clipping the natural pig casings, 20 additional vital *A. alata* mesocercariae were spiked to the filling (batch 1) or not (batch 2) Drying: 26 °C, RH: 40-60%, 7 days | Hams: average weight 195 g; initial pH: 5.8±0.2; final pH: 5.3±0.2 Salami: average weight 100 g; initial pH: 5.5±0.2; final pH: 4.6±0.1 Raw/“Knackwurst” sausages: average weight 70 g; initial pH: 5.5±0.1; final pH: 5.0±0.2 | *Alaria alata* | *Alaria alata* mesocercariae migration technique (AMT) for the presence of vital and non-vital mesocercariae | 100% of *A. alata* mesocercariae were inactivated during the traditional raw ham production.  5/42 salami sausages (11.9%) and 4/22 “Knacker” sausages (18.2%) contained vital *A. alata* mesocercariae after a maturation period of at least 24 h but no further vital parasites were observed in any of the sausage samples. | **Gonzales-Fuentes et al., 2014** |
| Cured | **Yamanashi, Japan** | Blocks (n=8) of meat (5 × 4 × 3 cm; 50 g) infected with *Sarcocystis*, trimmed of fat and connective tissue, from Japanese sika deer (*Cervus Nippon centralis*). | Each 50 g block of meat (5 × 4 × 3 cm) was soaked or rubbed with 2.0 or 6.0% NaCl and/or nitrite- enriched curing salt (NCS = NaCl enriched with 5% sodium nitrite, 10% potassium nitrate) and stored at 4°C for up to 7 days. | NA | *Sarcocystis spp.* | Digestion method for the purification of *Sarcocystis* bradyzoites | *Sarcocysts* parasite were inactivated within 1 day by the following combinations: 6.0% salt and 2.0% NCS; 2.0% salt and 0.25% NCS; 6.0% salt only; and 2.0% salt only.  However, <1.0% NCS alone failed to inactivate bradyzoites within 7 days.  These findings indicate that more than 2.0% salt and NCS were effective in reducing the viability of *Sarcocystis spp.*. | **Honda et al., 2018** |
| Cured | **Poland** | 3 industrially produced batches (200 kg each) of raw-aged wild boar loin (no fat/skin); 3 treatments; sampled at 0, 14, and 28 days. | Three treatments;  Treatment C (control) - curing salt (99.5% NaCl, 0.5% NaNO2; 1.6% of weight of the meat). Left to cure for 48h at 4°C.  Glucose (5g/kg) + water (4% by weight of meat) applied.   Treatment R1 - curing salt (99.5% NaCl, 0,5% NaNO2; 1.6% of weight of the meat) Left to cure for 48h for 4°C.  Glucose (5g/kg) and apple vinegar (4% by weight of meat) then applied.   Treatment R2 - curing salt (99.5% NaCl, 0.5% NaNO2; 1.6% of weight of the meat) + apple vinegar (4% by weight of meat). Left to mature for 48h at 4°C Glucose (5g/kg) applied.  Loins were suspended on smoking sticks.  Maturing process 15–17 °C and humidity of 75–80% for 2-3 days Cold smoking at 20–25 °C for 1–1.5 hours. Maturation continues for 4 weeks with the assumed initial parameters of the process and low air movement.  Products were vacuum-packed and placed in a cooling room (4 ◦C). | Approx. 35% weight loss    pH values Treatment Day 0 Day 14 Day 28 Control 5.50±0.17 5.70±0.04 5.35±0.20 R1 5.36±0.13 5.24±0.02 5.10±0.04 R2 5.17±0.19 5.33±0.01 5.34±0.08 | Coagulase-positive staphylococci *Salmonella* spp. *Listeria* spp. *Campylobacter* spp. | Classical culture-based methods - ISO standard microbiological methods | Coagulase-positive staphylococci were found in the control sample at day 0, day 14 and day 28 (2.05, 1.85 and <1.0 log cfu/g, respectively)  *Staphylococcus aureus*, *Salmonella* spp., *Listeria* spp. and *Campylobacter* spp. were not found in any of the samples tested. | **Łepecka et al., 2023** |
| Cured | **Italy** | 59 samples of game meat from four wild species (alpine chamois (*Rupicapra rupicapra*), roe deer (*Capreolus capreolus*), red deer (*Cervus elaphus*) and wild boar (*Sus scrofa*) at different production stages from four different processing plants | NA | Product aw (mean) pH (mean) Raw game meat 0.98 5.7  Fresh sausage 0.94 5.4 Cured sausage 0.85 5.7 | *Listeria monocytogenes* | ISO standard microbiological method API® Listeria BioMerieux for identification Serotyping by conventional serological method and multiplex PCR. Subtyping by PFGE. | 76% of analysed samples (45/59) tested positive for *Listeria* spp. Of these 14 (24%) were classified as *L. monocytogenes* and 31 (52%) as other *Listeria* species.  *L. monocytogenes* was present in 38% of the ready-for-sale cured sausages but under the legal limit of 100 cfu/g and consistently under the method’s detectable limit (10 cfu/g). *L. monocytogenes* strains isolated from game meat products belonged to serotype groups other than group-2 apart from a strain from wild boar sausages from one plant | **Lucchini et al., 2014** |
| Fermented | **Croatia** | Three lots of wild boar meat sausages (WB) and three deer meat sausages (DS) (1:1 ratio with pork) originating from 5 commercial sausage producers. n = 105: triplicate samples from each of the sausage producers at days 0, 4, 7, 10, 20, 40 and at the end of the ripening period (day 20 or 40). | Mixture of wild boar (*Sus scrofa*) or deer (*Cervus elaphus*) meat with pork (*Sus scrofa domesticus*) in 1:1 ratio. Additional ingredients: salt (2.0–2.2 %), white wine (1%), fresh garlic (0.3–0.4%), ground red chili peppers (0.3%), ground black peppers (0.1–0.2%) and ground red sweet peppers (0.1%). No starter cultures or nitrites.  RH 52−92%; -3−15 °C; cold intermittent smoking first two weeks. | Sausages 650 g in collagen casings (d = 35 mm) and 550 g in natural casings (d = 38 mm). Day 0: aw: 0.95−0.97; pH: 5.54−5.64  Day 4: aw:0.93−0.95−; pH: 5.41−5.60  Day 7: aw: 0.92−0.94; pH: 4.99−5.54  Day 10: aw:0.90−0.93; pH: 4.88−5.55  Day 20: aw: 0.83−0.97; pH: 4.89−5.51  Day 40: aw: 0.84−0.87; pH: 5.04−5.53 | *Bacillus cereus* group *Listeria monocytogenes* *Salmonella* spp. *S. aureus* | Classical culture-based methods - ISO standard microbiological methods. | End-products (log cfu/g)  *Bacillus cereus* group <1.0−5.7 *Listeria monocytogenes* not detected in the end products (detected during fermentation and ripening of WB sausage,< 2 log cfu/g) *Salmonella* spp. not detected *S. aureus* <1.0 (sporadically presented up to 20 days) | **Maksimovic et al., 2018** |
| Cured | **USA, California** | Outbreak linked to venison jerky. | NA | NA | *Clostridium botulinum*, type F | Toxin was extracted from the jerky using a gelatin-phosphate buffer and tested in mice. Meat and curing ingredients were tested for *C. botulinum* after anaerobic incubation at 30°C | No toxin or organisms were found in the ingredients, but toxin was repeatedly detected in the jerky. | **Midura et al., 1972** |
| Fermented | **Italy, Campania** | A total of 162 samples were tested: 99 pork products (including raw bacon, cold cuts, salami, and raw cured meats) and 63 wild boar sausages/salami. | Pork products were bought from a local market in Campania; wild boar products were homemade. Both were cured, though curing details were unspecified. | NA | *Hepatitis E virus* (HEV) | Sample preparation according to Szabo et al. (2015) | RNA extraction from 41 samples had <1% recovery and were HEV-negative. Upon retesting, 4 wild boar and 1 pork sausage were RT-qPCR positive; the pork sausage later tested negative, possibly due to low or uneven contamination. Only 4 of 63 (6.3%) wild boar salamis—made without liver—were HEV-RNA positive. All 39 uncured pork products tested negative. | **Montone et al., 2019** |
| Fermented | **Croatia** | 8 treatments × 3 batches (n=24) of ripened sausages; each batch: 5 kg pig (S*us scrofa domesticus*)/wild boar (S*us scrofa*) meat mix | **Meat Composition:** 60% domestic pig, 40% wild boar  **Spices (%):** Salt 1.9, chili 0.5, garlic 0.3, sweet pepper 0.2, sugar 0.2, black pepper 0.1 **Starter Cultures:**  **B1–B3:** Native strains (LS0713, LS0296, LM0532 – single/combined)  **B4:** Control (no starter)  **B5:** Commercial (*L. sakei* + *S. carnosus*)  **B6–B8:** Encapsulated native strains (as B1–B3)    **Encapsulation Medium:** Sterile skim milk  **Casing:** Natural (pig intestine, 38 mm) **Fermentation/Ripening:** 40 days, 4 smoking treatments **Monitoring:** Temp/RH every 30 min **Sampling (n=72):** Days 0, 7, 40 (pH, water activity) | aw pH Treatment B1  Day 0 0.96 ± 0.00 5.58 ± 0.01 Day 7 0.95 ± 0.00 5.07 ± 0.01 Day 40 0.88 ± 0.01 5.11 ± 0.02 Treatment B2  Day 0 0.96 ± 0.00 5.56 ± 0.01 Day 7 0.95 ± 0.00 5.22 ± 0.01 Day 40 0.88 ± 0.01 5.31 ± 0.02 Treatment B3  Day 0 0.97 ± 0.00 5.55 ± 0.02 Day 7 0.95 ± 0.00 5.14 ± 0.00 Day 40 0.88 ± 0.01 5.15 ± 0.02 Treatment B4  Day 0 0.96 ± 0.00 5.59 ± 0.02 Day 7 0.95 ± 0.00 5.50 ± 0.01 Day 40 0.87 ± 0.01 5.43 ± 0.02 Treatment B5  Day 0 0.96 ± 0.00 5.58 ± 0.01 Day 7 0.95 ± 0.00 5.00 ± 0.00 Day 40 0.88 ± 0.01 5.03 ± 0.02 Treatment B6  Day 0 0.96 ± 0.00 5.57 ± 0.01 Day 7 0.95 ± 0.00 5.50 ± 0.00 Day 40 0.88 ± 0.01 5.48 ± 0.02 Treatment B7  Day 0 0.96 ± 0.00 5.56 ± 0.01 Day 7 0.95 ± 0.00 5.36 ± 0.02 Day 40 0.89 ± 0.01 5.43 ± 0.02 Treatment B8  Day 0 0.96 ± 0.00 5.57 ± 0.02 Day 7 0.95 ± 0.00 5.21 ± 0.01 Day 40 0.89 ± 0.01 5.49 ± 0.03 | *S*. *aureus* *Salmonella* spp. *L*. *monocytogenes* | Classical culture-based methods - ISO standard microbiological methods | *S*. *aureus* and *Salmonella* spp. not detected in any treatment at any time.  *L*. *monocytogenes*: Present in all treatments except **B1** (absent at day 7 and in final product). | **Mrkonjic Fuka et al., 2021** |
| Cured-Fermented | **Italy** | Bresaola.  Beef and horsemeat from S-America --> frozen ready-cuts Deer from New Zealand farms --> on the bone and frozen Wild boar from extensive farming system in Eastern-Europe --> on the bone and frozen Goat meat from farms in France --> on the bone and frozen  Samples were checked raw, as relatively cured, fermented and dried - varying only on the curing and fermenting time.  For each animal species, six samples were checked raw. 300 g from each animal were vacuum packed and transported in refrigerated containers to analysis. Final products were produced from the same lots of raw material; six samples from each animal species were taken at the end of the curing, fermenting and drying. | Frozen cuts were steam-thawed until internal temperature reached 4°C.  Cuts (deboned, when necessary) tumbled in a curing mixture (for 100 kg of meat: 2.5 kg of sodium chloride, 120 g of dextrose, 20 g of sodium nitrite, spices) at 2–4°C and left for 5–7 days at 3–4°C.  Initial temperature 22°C and RH 90%, after 24h temperature 18°C and RH 80%.  Two drying stages: 17°C and RH 80% for 15-30 days, then 14°C and RH 72% for 60-120 days. | Weight loss of the product was 45-50%.  Values of pH and aw for raw material and final products of different animal species (mean ± SD of six samples) Mean ± SD   **Raw material** pH Bovine 5:97 ± 0:07 Boar 5.75 ± 0:11 Deer 5:59 ± 0:03 Goat 6:27 ± 0:45 Horse 5:92 ± 0:06  aw  Bovine 0:96 ± 0:01 Boar 0:97 ± 0:01 Deer 0:96 ± 0:01 Goat 0:94 ± 0:01  Horse 0:94 ± 0:01  **Final product** pH  Bovine 6:72 ± 0:09 Boar 6:30 ± 0:05 Deer 6:05 ± 0:04 Goat 6:48 ± 0:04 Horse 5:81 ± 0:15 aw  Bovine 0:95 ± 0:01 Boar 0:90 ± 0:01 Deer 0:90 ± 0:01 Goat 0:88 ± 0:01 Horse 0:90 ± 0:02 | Pathogenic staphylococci *Clostridia Salmonella spp. Listeria monocytogenes* | Classical culture-based methods | Raw samples: *S. aureus* present in all samples of raw material.  No samples showed presence of *Salmonella, L. monocytogenes* or Clostridia,.  Pathogenic staphylococci were not isolated in the end game meat products. | **Paleari et al., 2002** |
| Fermented | **Serbia, Zlatibor** | Nine wild boars. From the meat was processed 40kg of ham and 50kg of sausages.  The remaining wild boar products were seized from consumers homes during the outbreak. | NA | NA | *Trichinella britovi* | *Trichinella* larvae were detected in the samples of the dried wild boa r meat, using the magnetic stirrer method. *Trichinella* species identification by polymerase chain reaction (PCR) analysis. | Artificial digestion; Dry muscle; 0,18 lpg (*Τrichinella* larva per gram) Sausages; 0,87 lpg.   PCR species identification revealed a single strain of *T*. *britovi* | **Pavic et al., 2020** |
| Fermented | **France, Allevard in the Belledonne massif of the French Alps** | Meat from a wild boar hunted in December 2021 | Salted raw wild boar meat hunted few months prior being ingested, hind leg salted and dry to make raw ham. Ham was consumed after 13 months | NA | *Trichinella britovi* | Artificial digestion | Detection of 8.32 lpg | **Peju et al., 2023** |
| Cured | **Umbria, Italy** | Traditional salami made from adult roe-deer (*Capreolus capreolus*) meat | Minced roe-deer and pork meat mixed with salt (2.2%), pepper powder and pepper grains (0.2%), garlic (0.05%) and starter cultures (a mix of *Staphylococcus xylosus* and *Staphylococcus carnosus* + *Lactobacillus sakei* in a 2:1 ratio). No addition of antioxidants and preservatives.  Meat was stuffed into a previously rehydrated dry-salted natural swine intestine. Following 10 days of drying inside hot chambers (22°C and 62% relative humidity (RH) for 48 h; 19°C and 66% RH for 76 h, followed by a 1°C temperature reduction and 1% increase in the RH each day, so as to reach 15°C and 72% RH within 10 days), the products were ripened in controlled seasoning rooms at 13°C and 75% RH for 60 days. | Immediately after stuffing (T0) and at 7 (T1), 14 (T2), and 60 days (T3), five samples tested from each product (SL - 33% roe-deer meat and 67% pork meat - low percentage, SH -50% roe-deer meat and 50% pork meat-high percentage , SC - 100% pork meat). pH: >5 after casing pH: ≈ 6 in final products  aw not influenced by product formulation | Sulphite-reducing bacteria *Listeria monocytogenes* *Salmonella* spp. | ISO standard microbiological methods | No detection of *L. monocytogenes* and *Salmonella* spp. in any sample at any time point. Sulphite-reducing bacteria (< 2 log CFU/g) detected in products after casing (T0) and in some salami samples. | **Ranucci et al., 2019** |
| Fermented | **Umbria, Italy** | Adult wild boars were hunted from May 2018 to May 2019 | Recipe: wild boar meat was combined with swine cuts and grinded (6mm fragments), mixed with salt (2.2%), black pepper grains (0.2%), garlic (0.05%) and starter cultures (a mix of *Staphylococcus xylosus* and *Staphylococcus carnosus* + *Lactobacillus sakei* in a 2:1 ratio), Antioxidants and preservatives were not used products were subjected to a drying process inside hot chambers for 10 days with different temperature (T) and relative humidity (RH) as follows: 22 °C and 62% RH for 48 h, 19 °C and 66% RH for 76 h followed by 1 °C T reduction and 1% RH increase every 24 h until reaching 15 °C and 72% RH. Afterwards, the salami were ripened in controlled seasoning rooms at 13 °C and 75% RH for 60 days | Analytical determination was performed immediately after stuffing (T0) and after 7 (T1), 14 (T2), and 60 days (T3) samples from each experimental group (Bl = 40% wild boar meat, BH = 60% wild boar meat, C= 100% pork) were collected and transported to the laboratory under refrigeration condition, aw was measured at 25 °C, aw values gradually decrease during seasoning time from T0 to T3, the pH of all the products decreased as storage progressed from T0 to T1, no significant differences between the experimental group were registered. | *Listeria monocytogenes  Salmonella* spp. | ISO standard microbiological methods | Absence of *L. monocytogenes* in all samples regardless of the product's composition and the sampling time. Presence of *Salmonella* serovar Typhimurium in the meat batter and in T1 salami samples of the group BH of batch 1, however, the pathogen was not detected in salami sampled at T2 and T3 time points.  *Salmonella* serovar Rissen was found in the meat batter of group BH from batch 4, but the microorganism was no longer present (absent in 25 g) in salami samples at T1, T2, and T3. | **Roila et al., 2021** |
| Fermented | **Namibia** | Three different salami batches with frozen raw meat from springbok (*Antidorcas marsupialis*), gemsbok (*Oryx gazella*), kudu (*Tragelaphus strepsiceros*) and zebra (*Equus burchelli*) that were shot by a professional harvesting team and purchased from an approved Namibian commercial meat wholesaler. | Each batch size was approximately 53 kg, comprising game meat (35.5 kg), pork back fat (14 kg), ground red pork skin (1.5 kg) and spices mix (2.1 kg).  Spices mix: 1.5 kg sodium chloride, 12.5 g sodium nitrate E251, 75 g ground black pepper, 12.5 g garlic, 50 g ground green cardamom, 100 g ground juniper berries, and 400 g cervelat, a maturing agent containing spices with mustard and hydrolysed vegetable protein (flavour enhancer), sodium ascorbate E301, 3.5% potassium nitrate E252 and dextrose.  Starter culture: *Pediococcus pentosaceus* and *Staphylococcus carnosus*.  Fermentation for 12 h at 18–22 °C and RH 92%.  Cold smoking with oak wood chips for 36 h at 22–24 °C and RH 92%.  Ripening: for a further 21 days at 12–16 °C and RH 75%. Vacuum packaging and storing at 4°C until analysis approximately 3 weeks after manufacturing. | Salami size (natural casings): diameter ± 75 mm and length ± 18 cm. Gemsbok salami aw: 0.79±0.007, pH: 5.00±0.063  Kudu salami aw: 0.81±0.018, pH:4.98b±0.067  Springbok salami aw: 0.82±0.013, pH: 5.46±0.034 Zebra salami pH: aw: 0.83±0.001, pH:4.98±0.023 | S*taphylococcus aureus* | Classical culture-based methods | Raw meat (mean values, log cfu/g): *S. aureus* < LOQ   End-products (log cfu/g): Gemsbok matured salami *S. aureus* < LOQ  Kudu matured salami  *S. aureus* < LOQ  Springbok matured salami  *S. aureus* < LOQ  Zebra matured salami  *S. aureus* < LOQ | **Schalkwyk et al., 2011** |
| Cured | **Italy** | Leftover dried-cured sausages (n=2) from meat of wild boars (n=2) that were not sampled for *Trichinella* spp*.* testing during post-mortem inspection | NA | NA | *Trichinella britovi* | Μagnetic stirrer artificial digestion Multiplex PCR | 15 and 17 lpg of meat, respectively, in the two sausage samples tested | **Turiac et al., 2017** |
| Cured | **KwaZulu-Natal, South Africa** | One sample (at least 20 g) of muscle (diaphragm), kidney, liver, heart, lung and lymph nodes (mandibular and parotid) collected from each of the carcasses of 7 African buffalo (*Syncerus cafer*) o and 7 greater kudu (*Tragelaphus stepsiceros*) with tuberculous lesions (n = 112).  For every carcass, an additional sample from the muscle tissue was used for the drying process experiment. | The biltong-making entailed cutting meat strips (20 g of each of the 14 animals), curing (12–18 hours) in a standard game biltong mixture (salt, sugar, vinegar and spices), subsequent drying and weekly monitoring. | NA | *Mycobacterium* spp. | Ziehl-Neehlsen staining of 10 week old colonies  PCR | No mycobacteria were isolated from any of the 14 tissue samples subjected to the biltong-making process (drying), though in the untreated pooled control muscle tissue sample (not subjected to the biltong making process) *M. bovis* was detected by PCR. | **Van der Merwe et al., 2010** |

NA: information not available

RH: Relative humidity

RTE: Ready-to-eat

LOQ: Limit of quantification
